# Perivascular Matrix Densification Dysregulates Angiogenesis and Activates Pro‐Inflammatory Endothelial Cells

**DOI:** 10.1002/advs.202510448

**Published:** 2026-06-17

**Authors:** Jingyi Xia, William Y. Wang, Kyle A. Jacobs, Kairav Maniar, Daphne Lin, Evan H. Jarman, Daniel L. Matera, Kristen Loesel, Christopher D. Davidson, Harrison L. Hiraki, Xiaotian Tan, Eve H. Shikanov, Robert N. Kent, Carole Parent, Xudong Fan, Ariella Shikanov, Matthew L. Kutys, Brendon M. Baker

**Affiliations:** ^1^ Department of Biomedical Engineering University of Michigan Ann Arbor Michigan USA; ^2^ Department of Cell and Tissue Biology University of California San Francisco San Francisco California USA; ^3^ Department of Chemical Engineering University of Michigan Ann Arbor Michigan USA; ^4^ Department of Cell and Developmental Biology University of Michigan Ann Arbor Michigan USA; ^5^ Department of Pharmacology University of Michigan Ann Arbor Michigan USA; ^6^ Rogel Cancer Center Michigan Medicine University of Michigan Ann Arbor Michigan USA; ^7^ Department of Macromolecular Science & Engineering University of Michigan Ann Arbor Michigan USA; ^8^ Department of Obstetrics and Gynecology University of Michigan Ann Arbor Michigan USA

**Keywords:** adherens junctions, angiogenesis, bleomycin, cell migration, endothelial cells, endothelial‐mesenchymal transition, extracellular matrix, fibrosis, microphysiologic systems, TGF‐β signaling

## Abstract

The potential for fibrosis across most organ systems may stem from connections to wound healing and the widespread presence of vascular endothelium. Endothelial cells (ECs) and angiogenesis have been heavily implicated in many organ‐specific fibrotic conditions, but little has been established in terms of how EC phenotype governs tissue healing vs. fibrosis. Here, we examined a murine lung injury model enabling EC lineage tracing and observed the invasion of aberrant ECs from the bronchial microvasculature following injury, along with concurrent densification of surrounding extracellular matrix fibers. To investigate mechanisms governing their appearance, we established a microphysiological system of human microvessels embedded within a tunable stromal matrix and found that heightened fiber density drives endothelial to mesenchymal transition to promote aberrant tip EC (ATEC) invasion into the matrix. ATECs remained adherent to fibrotic matrix and possessed a pro‐inflammatory phenotype that secretes TGF‐β2. Mechanistically, we identify ATEC formation was gated by destabilization of EC adherens junctions upon adhesion to fibrous matrix and associated regulation of TGF‐β signaling through a novel VE‐cadherin – TGF‐βR2 interaction. Altogether, this work identifies how enhanced fiber density associated with fibrogenesis regulates EC phenotype to generate pro‐inflammatory ATECs and suggests new contributions of ECs to fibrotic progression.

## Graphical abstract

Perivascular matrix densification promotes the emergence of aberrant endothelial tip cells (ATECs) that invade and persist within fibrotic microenvironments. Using in vivo lineage tracing and a human microvessel model, this study shows that fibrous matrix cues destabilize VE‐cadherin–mediated junctions to gate TGF‐β signaling, inducing a pro‐inflammatory endothelial phenotype that may contribute to lung fibrosis.

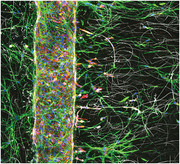

## 1 Introduction

Fibrotic tissue remodeling across many tissues and organ systems is often characterized as a persistent or excessive wound healing response. While myofibroblasts (MF) have been regarded as the direct cellular driver of fibrotic matrix remodeling and therefore primary focus of therapeutic targeting to date, accumulating evidence has highlighted the role of other cell types including endothelial cells (ECs), epithelial cells, and immune cells including macrophages as additional contributors [1]. ECs and the angiogenic process are central to wound healing; as such, it is plausible that changes to the extracellular matrix (ECM) in a fibrotic microenvironment may derail the EC angiogenic response from acting to restore tissue homeostasis to instead promoting disease‐driving cell function and associated signaling.

Supporting the involvement of ECs in fibrosis, histological examination of various murine models of fibrosis has consistently revealed microvascular abnormalities, but the direction (increased vs. decreased vascularity) [2, 3, 4], underlying mechanisms, and role of these changes in driving fibrosis have not been established. In vivo lineage tracing in various murine models of fibrotic disease (e.g. cardiac, cancer, lung, liver, and kidney) suggest as many as 30% of myofibroblasts may originate from EC precursors that undergo endothelial to mesenchymal transition (EndMT) prior to myofibrogenic differentiation [5, 6, 7, 8, 9, 10, 11]. Additionally, angiocrine signaling from ECs have been shown to control a switch between tissue regeneration vs. fibrotic progression in acute injury models of lung and liver fibrosis [12, 13, 14]. Fibrosis consistently involves progressive increases in matrix density and crosslinking which alter or impair parenchymal cell function, ultimately leading to organ failure. However, it remains unclear if and how these biophysical changes to the ECM influence EC signaling and function after tissue injury and in fibrotic diseases.

Observations consistent across in vivo and in vitro models of angiogenesis support a critical, initiating step of EC activation followed by the invasion of tip cells into the surrounding interstitial matrix that lead multicellular sprouts to extend capillaries. Given clear parallels to epithelial cell invasion enabled by epithelial‐mesenchymal transition, previous work has implicated EndMT in the initiation of angiogenesis [7, 15, 16, 17, 18]. A separate body of work suggests ECs can directly contribute to fibrosis in vitro and in multiple organ systems via EndMT and subsequent myofibrogenesis [10, 11, 19, 20, 21, 22, 23, 24, 25, 26]. The established central role of MFs along with evidence that EndMT induces a multipotent EC phenotype has led many to explore whether ECs drive fibrotic progression by transdifferentiating into bona fide MFs (i.e. possessing α‐smooth muscle actin^+^ F‐actin stress fibers). However, the varied identification of these cells in different organ fibroses despite consistent vascular abnormalities raises the question of whether ECs may drive fibrosis through alternative, yet equally important, mechanisms.

Previous work on EndMT has largely focused on biochemical and genetic mediators [10, 11, 19, 20, 21, 22, 23], however studies of epithelial‐mesenchymal transition indicate that physical ECM cues can potently modulate the impact of such signals [27, 28, 29, 30]. A parallel but currently unexplored concept may extend to EndMT and the angiogenic response, which is supported by observations that angiogenesis is highly sensitive to physical properties of the surrounding ECM [31, 32, 33, 34]. Interestingly, critical to angiogenesis is the dynamic regulation of vascular adherens junctions (AJs), which mechanically stabilize EC‐EC connections and directly regulate effector signaling pathways governing EC fate and behavior [35, 36]. Further, biochemical and mechanical cues mediate signaling at EC‐ECM adhesions which in turn influence the assembly and stability of EC AJs. Thus, dissecting the potential relationship between physical and soluble microenvironmental cues that may underlie EC fate and function requires integrative systems that can sufficiently replicate the native tissue microenvironment while providing experimental control.

Informed by observations in the murine bleomycin lung injury model, here we integrated tunable fibrous hydrogel composites and microphysiological systems to explore the influence of fibrotic matrix (i.e. heightened perivascular fiber density) on arteriole/venule‐scale microvascular endothelium and investigated how EC‐ECM interactions modulate the activation of quiescent microvessel ECs into invasive and aberrant tip ECs (ATECs). We demonstrate heightened fiber density via EC mechanosensing destabilizes EC AJs, diminishes vascular barrier function, and simultaneously increases ATEC formation via EndMT. Using unbiased proteomics, we identify fiber‐induced AJ destabilization decreases a VE‐cadherin – TGFβ‐R2 interaction which underlies fiber‐mediated enhancement of EC TGF‐β signaling. Further, transcriptomic and secretomic characterization of ATECs revealed that these cells transition toward a secretory, pro‐inflammatory phenotype, and are additionally a significant source of TGF‐β2, a key pro‐fibrotic growth factor that may act on macrophages, fibroblasts, but also ECs resident to parental vasculature [10, 21]. Indeed, exposure of parent vessel endothelium to TGF‐β2 leads to heightened EC apoptosis as a function of perivascular fiber density, consistent with observations of arteriolar/venular EC apoptosis in the bleomycin mouse model. Together, our studies describe how changes in perivascular ECM fiber density destabilize EC AJs, promote EndMT, and the formation of inflammatory and pro‐fibrotic ATECs. Furthermore, this work provides evidence for a fibrotic feedforward cycle mediated purely by ECs that may act in parallel or synergistically with myofibroblast driven fibrotic activity.

## 2 Results

### 2.1 Characterization of EC Expansion in a Murine Model of Pulmonary Fibrosis

**FIGURE 1:**
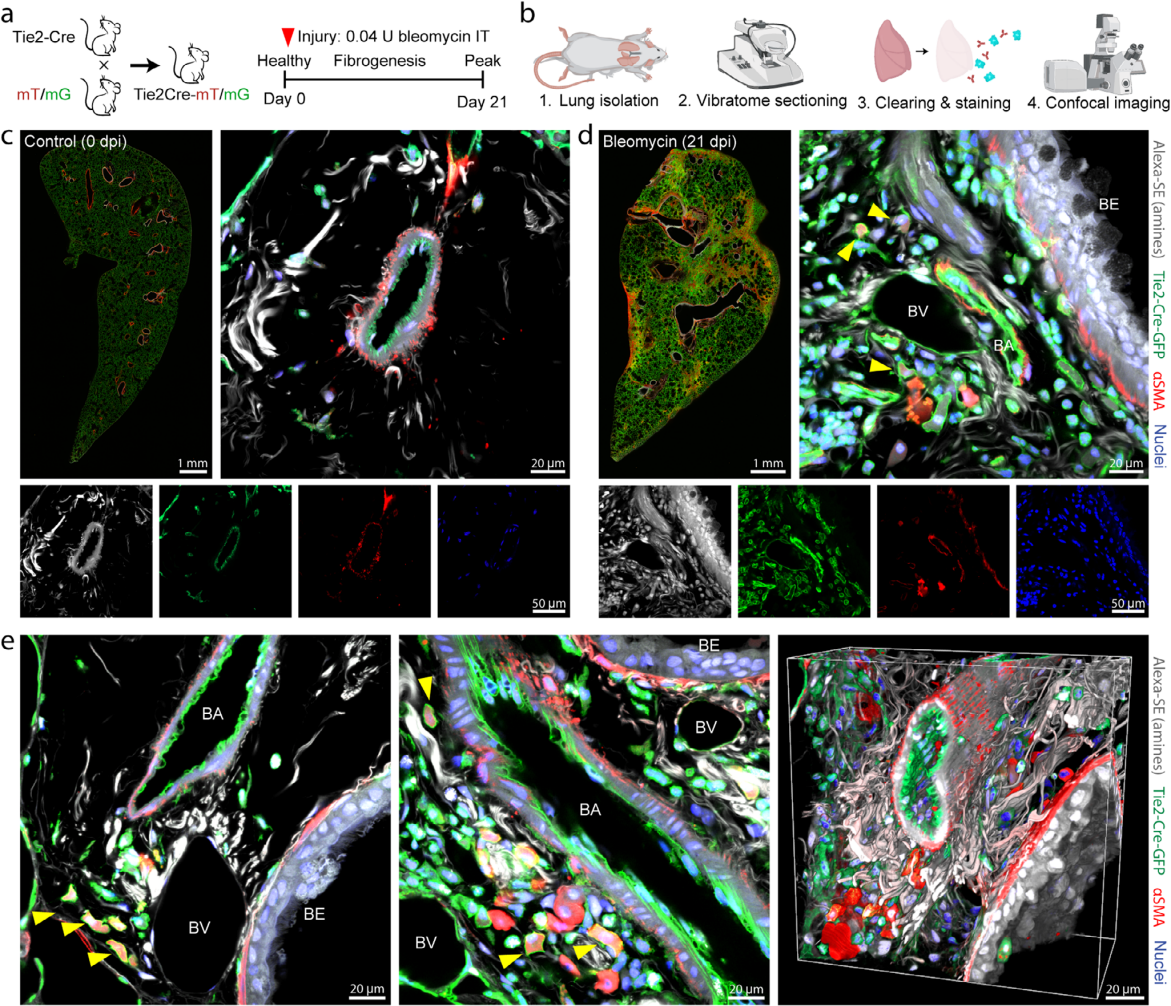
Emergence of aberrant ECs in the bleomycin lung injury model. (a) Breeding strategy and study timeline for EC lineage tracing during bleomycin injury‐induced lung fibrogenesis. (b) Pipeline for 3D imaging of lung tissue. Representative low magnification whole‐lobe tile‐scan images and high magnification single z‐slice images of peribronchial vasculature in (c) uninjured control lung and (d) bleomycin‐treated lung tissues 21 days post‐injury. Movie S1: same image set presented as a slice‐through movieshowing multiple vessels and abundant single ECs. (e) Representative high magnification single z‐slice images (left, middle) and 3D rendering (right) depicting cross‐sectional and longitudinal views of bronchial vasculature from day 21 bleomycin treated mice. Yellow arrow heads mark individualized Tie2‐Cre‐GFP positive cells expressing αSMA. BA: bronchial arterioles, BV: bronchial venules, BE: bronchial epithelium. Movie S2, S3: same image sets presented as slice‐through movies.

To track the location and morphology of pulmonary ECs during injury‐induced lung fibrogenesis, we developed a 3D lung tissue imaging pipeline combining a genetically engineered mouse model enabling EC lineage tracing, precision cut lung slices (PCLS, 200 µm thick), optical clearing methods, and volumetric confocal imaging. Adult male Tie2‐Cre/mTmG, where Tie2 (TEK)‐driven Cre recombinase activity drives a permanent switch from membrane‐localized tdTomato to GFP expression, were administered a single intratracheal instillation of bleomycin (0.04 U/mouse) to induce lung injury and initiate fibrotic remodeling (i.e. matrix deposition and crosslinking), which typically peaks in severity after 2–3 weeks. (Figure 1a). Following cardiac perfusion and lung extraction, PCLS were sectioned by vibratome and cleared using the CUBIC method [37], immunostained for α‐smooth muscle actin (αSMA), counterstained with DAPI and Alexa Fluor succinimidyl ester (AFSE, which fluorescently labels all amine‐containing ECM and cellular proteins), and imaged by laser‐scanning confocal microscopy (Figure 1b). Low magnification tile‐scan imaging of entire lung sections across healthy control (day 0) and tissues at the peak of the fibrotic response (day 21) revealed marked changes in GFP^+^ EC and αSMA^+^ MF density resulting from bleomycin‐induced lung injury. In control lungs, EC density within alveolar regions was uniform and αSMA expression was restricted to smooth muscle cell linings of airways and larger‐scale vasculature (Figure 1c). In contrast, bleomycin injured lungs revealed heterogeneously distributed regions of densified ECs and regions enriched for αSMA^+^ MFs both proximal to central bronchial airways as well as in the distal lung (Figure 1d and Movie S1).

Higher magnification confocal imaging revealed intriguing changes specific to the bronchial microvasculature (50–200 µm diameter, thereby excluding capillaries) and the perivascular ECM supporting these vessels (Figure 1d,e and Movies S2 and S3). Bronchial arterioles (BA, identified by the presence of an α‐SMA^+^ smooth muscle cell lining in large vessels proximal to bronchi) consistently possessed a surrounding fibrous matrix, as visualized by AFSE‐labeling, which typically radiated outward from the BA. Bronchial veins (BV) appeared proximal to BAs, lacked smooth muscle cell investment, and typically possessed a markedly thinner vessel wall [38]. This structural difference likely reflects the distinct pressures experienced by the bronchial arterial and venous systems. Bronchial arterioles originate from the high‐pressure system circulation and therefore contain smooth muscle and adventitial support that enable them to withstand pulsatile blood flow and regulate vascular tone. In contrast, bronchial venules return blood at much lower pressure. Three weeks after bleomycin‐induced injury, perivascular fiber density increased around BA/BVs and numerous GFP^+^ ECs were found to be embedded within this densified ECM. Interestingly, these ECs did not constitute multicellular sprouts typically associated with angiogenesis, but instead appeared as dispersed, individualized cells lacking intercellular connections. A subpopulation of these ECs found proximal to BAs/BVs were additionally α‐SMA^+^ (Figure 1d,e, yellow arrows), suggestive of EndMT, although high magnification imaging revealed an absence of α‐SMA localization to stress fibers and instead diffuse staining throughout the cytosol. As macrophages have also been shown to express Tie2 [39, 40], we immunostained for pan‐monocyte/macrophage marker F4/80 and confirmed that the majority of GFP^+^ cells (including those that were dually GFP^+^ and αSMA^+^) were not macrophages (Figure S1 and Movies S4 and S5).

Overall, these studies indicate that following lung injury, the density of perivascular matrix fibers increases surrounding arteriole/venule‐scale bronchial vasculature and concurrently, a population of aberrant ECs that lack intercellular connections appear and adhere to the perivascular matrix. Whether these two observations are linked, and if so, how ECM fiber density may regulate EC signaling and behavior remain unknown. Given the high degree of spatiotemporal heterogeneity in tissue response to bleomycin‐induced injury and the fact that there are no means to directly modulate ECM fiber density in vivo, we turned to in vitro biomaterial and microphysiological system (MPS) approaches to answer these questions.

### 2.2 Heightened Matrix Fiber Density Induces Formation of Aberrant ECs in the Absence of Soluble Cues

**FIGURE 2:**
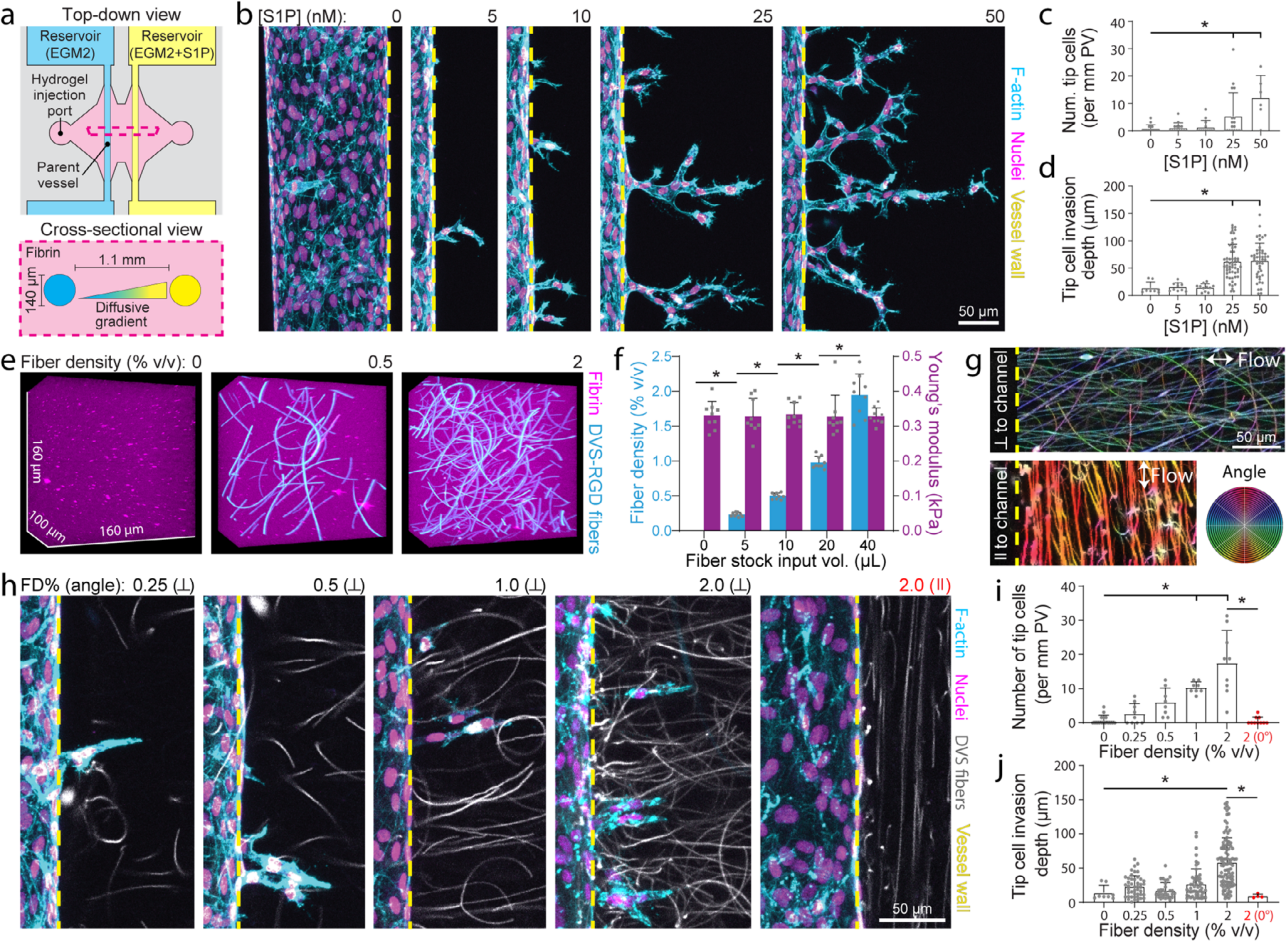
Heightened matrix fiber density induces spontaneous tip cell formation in the absence of soluble cues. (a) Schematic of microvessel microphysiological model and tip cell formation assay. (b) S1P‐induced tip cell formation over 4‐days within 10 mg/ml fibrin hydrogels as a function of S1P concentration. Nuclei (magenta), F‐actin (cyan), yellow dashed lines indicate parent vessel edge. (c) Quantification of number of tip cells (data representative of N>3 replicate studies with n>5 fields of view analyzed per condition) and (d) tip cell invasion depth as a function of [S1P] (data representative of N>3 biological replicates with n>7 invading tip cells per group). (e) 3D rendering of fiber‐embedded fibrin hydrogels at varying fiber density. Synthetic DexVS fiber segments (cyan), fluorescently labeled fibrin hydrogel (magenta). (f) Quantification of fiber density and Young's modulus as a function of input fiber stock volume. N > 3 individual hydrogels per group. (g) Flow‐induced fiber alignment perpendicular (0°) or parallel (90°) to the long axis of the parent vessel. (h) Fiber‐induced tip cell formation (without S1P added to the chemokine channel) over 4‐days within 10 mg/ml fibrin hydrogels and indicated fiber density (FD). Nuclei (magenta), F‐actin (cyan), fibers (white), yellow dashed lines indicate parent vessel edge. (i) Quantification of number of tip cells (data representative of N>3 replicate studies with n>8 fields of view analyzed per condition) and (j) tip cell invasion depth as a function of fiber density (data representative of N>3 replicate studies with n>4 invading cells analyzed per condition). Blue indicates 90° fiber alignment. All experiments were conducted with HUVECs. All data presented as means ± standard deviations; * indicates a statistically significant comparison with *p*<0.05 (one‐way analysis of variance).

To test whether perivascular matrix fiber density modulates a switch between EC quiescence vs. activation and subsequent invasion, we integrated a previously established multiplexed MPS containing perfusable arteriole/venule‐scale microvessels [41, 42, 43] with tunable, hybrid natural‐synthetic hydrogel‐fiber composites previously established by our group for modeling interstitial or stromal matrix [44, 45, 46]. Each MPS contains two parallel microchannels (Ø = 140 µm) fully embedded within a user‐defined hydrogel, with each channel terminating in individually addressable media reservoirs (Figure 2a). Here, we used fibrin (10 mg/ml), a naturally derived hydrogel commonly employed to model ECM during wound healing [47] which is known to leak from hyperpermeable vasculature and accumulate in the extravascular space during lung injury and fibrosis [48]. One of each pair of channels was seeded with ECs, which self‐assembled into a patent, arteriole/venule‐scale parent microvessel overnight (16 h post cell seeding) possessing VE‐cadherin‐enriched AJs and low vessel wall permeability [43].

Chemokines can be added to the adjacent, unseeded channel to generate a diffusive chemoattractant gradient that drives endothelial tip cell formation and subsequent angiogenic sprouting (Figure 2a) [41, 42, 43]. To confirm that this platform enables a robust, quantitative assessment of tip cell formation, we introduced a well‐established EC chemoattractant, sphingosine‐1‐phosphate (S1P) [43, 49], to the chemokine channel at varying concentrations. Indeed, the number and invasion depth of invading tip cells increased as a function of S1P concentration and resulting gradient strength (Figure 2b–d), establishing a robust assay and quantitative metrics for subsequent studies. Our prior work demonstrates that balance between a chemokine gradient (S1P) and mitogen are required for multicellular sprout invasion leading to the formation of perfusable capillaries and that changes in matrix density can lead to the invasion of single ECs that fail to form functional vasculature [33, 43].

Individual microenvironmental cues presented by fibrin hydrogels are challenging to decouple and orthogonally tune (e.g. hydrogel stiffness, adhesive ligand density, and porosity all vary as a function of fibrinogen density). Furthermore, our above characterization of increased density of perivascular fibers revealed matrix fibers with multi‐micrometer diameters far greater than the <200 nm diameters characteristic of fibrin [50] (Figure 1). Thus, we implemented our recently established composite hydrogel approach where a natural or synthetic bulk hydrogel can be combined with chemically and mechanically defined synthetic fiber segments that possess the geometry and mechanics of collagen fibrils prominent in the interstitial ECM of patients with idiopathic pulmonary fibrosis and other forms of interstitial lung disease [45]. Cell‐adhesive fiber segments were generated via electrospinning of a synthetic polymer solution, dextran vinyl sulfone (DexVS), followed by segmenting fibers to defined lengths, and functionalization with the cell‐adhesive peptide RGD. DexVS fiber segments were then incorporated into the fibrin hydrogel precursor solution at controlled v/v % to define the fiber density (0–2 v/v %) of the resulting hydrogel composite (Figure 2e). Modulation of DexVS‐RGD fiber density did not alter the stiffness of the bulk fibrin hydrogel as measured by AFM nanoindentation (Figure 2f), but likely influences local, cell‐scale mechanics given that these fibers individually are considerably stiffer than bulk fibrin [42, 51]. In prior work employing this composite approach, we demonstrated that heightened fiber density promotes myofibroblast activation and fibrogenic activity of encapsulated fibroblasts, in contrast to simply increasing the density or crosslinking/stiffness of an amorphous bulk hydrogel that lacks fibrous microstructure [45]. Here, we employed this approach to examine whether perivascular matrix fiber density modulates the activation of quiescent ECs into invasive tip cells.

Additionally, to model the matrix fiber alignment characteristic of the perivascular matrix observed in our in vivo studies (Figure 1), we adopted a flow‐induced fiber alignment methodology previously utilized for aligning collagen fibrils within type 1 collagen matrices [52, 53]. To generate fiber alignment perpendicular to the long axis of the parent vessel (90° alignment) as we observed in vivo, the hydrogel precursor solution was injected orthogonal to inserted channel‐molding needles to generate a fluid flow profile that aligns fibers radially with respect to the eventual vessel (Figure 2g). To align fibers parallel to the long axis of the parent vessel (0° alignment), hydrogel precursor solution was first injected into the device and subsequent insertion of acupuncture needles resulted in fluid flow profiles that aligned the fibers in the direction of needle insertion (i.e. parallel to eventual parent vessels). Prior to cell seeding, microchannels were coated with basement membrane proteins (Matrigel) to establish equivalent initial matrix ligand composition and topography for ECs adhering to microchannel walls, despite variations in fiber alignment and density in the surrounding matrix. Indeed, 16 h after EC seeding, parent vessels in non‐fibrous control vs. fibrous fibrin hydrogels did not differ in vessel diameter, cell density, or permeability as assessed by fluorescently labeled dextran diffusion across the vessel wall (day 1 control condition shown in Figure 4a,b).

Angiogenic sprouting is typically driven by exogenous chemotactic gradients, such as VEGF, that drive EC activation and directed migration. To our surprise, however, we found that despite the absence of any exogenous soluble gradient, heightened perivascular fiber density (FD) was sufficient to drive an invasive EC phenotype. Over 3 days, we observed a significant increase in ECs detaching from the parent vessels as a function of fiber density (Figure 2h–j). These cells invaded the surrounding matrix as individual, disconnected cells. We termed them **aberrant tip ECs (ATECs)** because their morphology resembled the leading tip cells of an angiogenic sprout, but they completely lacked intercellular adhesion characteristic of multicellular sprouts. This finding suggests that physical cues from the matrix can independently promote EC activation and invasion. The formation of ATECs proved highly dependent upon fiber orientation with respect to the parent vessel, as an equivalently high density (2% v/v) of fibers, but aligned parallel to the parent vessel axis (0° alignment), resulted in virtually no EC invasion (Figure 2h–j). These results strikingly demonstrate that both fiber density and alignment of perivascular matrix fibers regulate the activation of ECs and their subsequent invasion into the surrounding matrix, drawing clear parallels to prior work on EMT and epithelial cell migration in the context of cancer metastasis [54, 55].

The studies above employed human umbilical vein ECs as a model EC due to their frequent use throughout the field of vascular biology. To assess whether ECs specific to tissues known to be susceptible to fibrosis would similarly respond to densified, radially aligned fibers surrounding an arteriole/venule‐scale vessel, we performed identical studies with primary human liver, lung, and dermal microvascular ECs. Heightened fiber density led to increased ATEC formation and invasion in liver and lung microvascular ECs (Figure S2a–c). However, dermal microvascular ECs did not activate or invade to any degree. While our synthetic matrix‐like fibers are chemically and mechanically defined, and thus highly tunable, their dextran‐based composition is non‐native to mammalian tissues; thus, we sought to confirm whether a similar phenomenon occurred in response to natural collagen fibers given that fibrotic tissue is rich in fibrillar collagen. To do so, we adopted a recently established approach to generating larger, micrometer‐scale collagen fibers that possess comparable diameters to collagen fibers observed in vivo (Ø > 1 µm), in contrast to those comprising traditionally prepared collagen hydrogels (Ø = 250–500 nm) [45, 53]. Micrometer scale collagen fibers were isolated and suspended within fibrin hydrogels and identical studies as described above demonstrated that collagen fibers also induced ATEC formation (Figure S3). Bronchial microvessels observed in vivo were thin‐walled with maximally a single layer of smooth muscle cells; however, perivascular cells including pericytes and smooth muscle cells promote vascular stability and their interaction with ECs can be disrupted during fibrogenesis. We therefore incorporated human mesenchymal stem cells (MSCs), which are widely used to model perivascular cells due to their transcriptional and functional similarities to pericytes and smooth muscle cells, to generate perivascular cell–invested microvessels. Despite the presence of perivascular cells, heightened fiber density still induced ATEC formation and interestingly led to perivascular cell divestment and polarized migration into the surrounding matrix as compared to non‐fibrous controls (Figure S4).

### 2.3 ATEC Formation due to Increased Interstitial Fiber Density Involves Matrix Mechanosensing and EndMT

**FIGURE 3:**
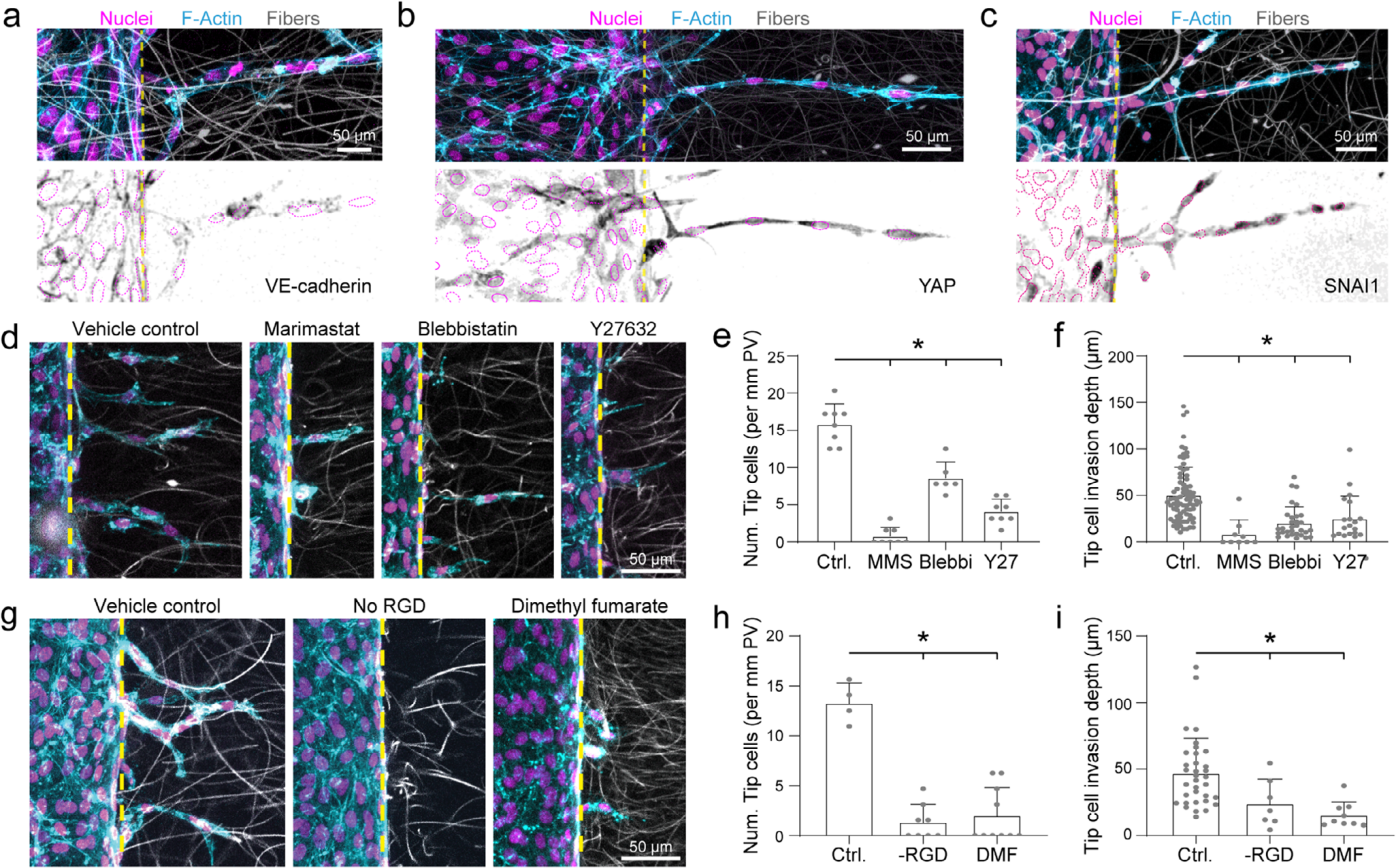
Fiber‐mediated ATEC activation and invasion share signaling and cellular functions previously implicated in EndMT and angiogenic tip cells. (a–c) Representative confocal images (maximum intensity projections) depicting the differential localization of immunostained VE‐cadherin (a), YAP (b), and SNAI1 (c) invading ECs and ECs within the parent microvessel (red arrow) after 4 days of culture. Top row: nuclei (magenta), F‐actin (cyan), DexVS‐RGD fibers (grey), yellow dashed lines indicate parent vessel edge. Bottom row: inverted grayscale images, magenta dashed lines indicate boundaries of nuclei, yellow dashed lines indicate parent microvessel edge. (d–f) Representative images (d) of vehicle control (DMSO 1:1000 dilution), marimastat (1 µm), blebbistatin (30 µm), or Y27632 (25 µm) treated microvessels (treated over days 1–4 of culture) within fibrin + FD 2% hydrogels after 4 days of culture, along with corresponding quantification of the number of invading ECs (e: N=3 replicate studies with n>6 fields of view analyzed per condition) and EC invasion depth (f: N=3 biological replicates with n>9 invading cells analyzed per condition. (g–i) Representative images (g) of vehicle control microvessels with RGD‐functionalized fibers (fibrin + FD 2%), control microvessels within fibrin + FD 2% hydrogel but where fibers that were not functionalized to enable cell adhesion (No RGD, and dimethyl fumarate (100 nm) treated microvessels with RGD‐functionalized fibers (fibrin + FD 2%) after 4 days of culture. (h,i) Corresponding quantification of the number of invading ECs (h: Data representative of N=3 replicate studies with n>4 fields of view analyzed per condition for number of tip cells (h) or n>7 invading tip cells per condition for cell invasion depth (i). Note that the number of invading tip cells in conditions lacking RGD or with DMF treatment were exceedingly low. All experiments were conducted with HUVECs. All data presented as means ± standard deviations; * indicates a statistically significant comparison with *p* < 0.05 (one‐way analysis of variance with Tukey's post hoc test).

EndMT has been previously implicated in the transition of quiescent vessel‐lining ECs into invasive tip cells [7, 11, 56] and in separate work, associated with EC mechanosensing of stiffened ECM [6]. However, prior studies primarily relied on EC cultures on 2D substrates with tunable stiffness. In contrast, angiogenesis is inherently a 3D process involving the proteolytic invasion of ECs through a surrounding fibrous ECM. Thus, we examined whether ECM fiber density similarly influenced EC mechanosensing and EndMT in our 3D model. Invading ATECs possessed thinner to nearly no AJs as evidenced by VE‐cadherin immunostaining, in contrast to the more robust maintenance of AJs in adjacent ECs within the parent microvessel (Figure 3a). Supporting an EndMT‐mediated activation of tip cells, SNAI1 (Snail) immunostaining revealed overall heightened cytosolic and nuclear SNAI1 localization in invading ECs compared to lower SNAI1 intensity in parent vessel ECs (Figure 3c). Consistent with these findings, ECs cultured on fibrous substrates increased their expression of EndMT‐associated genes (*SNAI1, SNAI2, CDH2*), while decreasing VE‐cadherin expression in contrast to controls on biochemically identical surfaces lacking fibrous topography (Figure S5). YAP immunostaining revealed similar expression levels and distribution to Snail. In the parent vessel ECs (Figure 3b), YAP appeared diffuse and was largely absent from nucleus, consistent with ECs in a quiescent state. In contrast, invading ATECs possessing higher overall levels of YAP and comparable distributions between cytosolic and nuclear compartments.

Toward identifying critical cellular requirements for the activation and invasion of ATECs, we utilized a panel of pharmacologic inhibitors targeting mechanosensing and EndMT‐associated cell functions. We tested requirements for matrix proteolysis and actomyosin contractility, both of which have been previously shown to be gained by ECs that have undergone EndMT [10]. Treatment with marimastat, a broad‐spectrum inhibitor of MMP‐mediated proteolysis, decreased both the number of ATECs and their invasion depth, as ECs were unable to degrade the surrounding matrix and generate sufficient space for invasion (Figure 3d–f). Treatment with a myosin II inhibitor (blebbistatin) or ROCK inhibitor (Y‐27632) decreased ATEC formation, in line with the premise that actomyosin contractility is a key requirement for cell migration in 3D and matrix mechanosensing (Figure 3d–f). As the protrusions of invading ECs were observed to extend along fibers (Figure 3a–c), we next tested whether direct integrin‐mediated adhesion to fibers was critical for ATEC invasion. Indeed, identical studies performed with fibers lacking RGD functionalization resulted in reduced ATEC numbers and invasion distances (Figure 3g–i), indicating that direct EC engagement to matrix fibers is a requirement for ATEC formation. Lastly, recent studies in addition to the studies described above have demonstrated that fibrous matrices can modulate YAP/TAZ signaling in EC monolayers to promote cell migration [57]. Providing further support for the involvement of YAP/TAZ signaling in EC activation and invasion in this setting, treatment with dimethyl fumarate, a potent although non‐specific inhibitor of the YAP/TAZ signaling pathway [58], decreased ATEC formation (Figure 3g–i). Overall, these studies establish that proteolysis, actomyosin contractility, cell‐matrix adhesion, and mechanosensing through YAP/TAZ are all required for the transition from EC quiescence to activation and invasion into a surrounding fibrous matrix

### 2.4 Fiber‐Induced AJ Destabilization Decreases Vessel Barrier Function and Promotes ATEC Invasion

**FIGURE 4:**
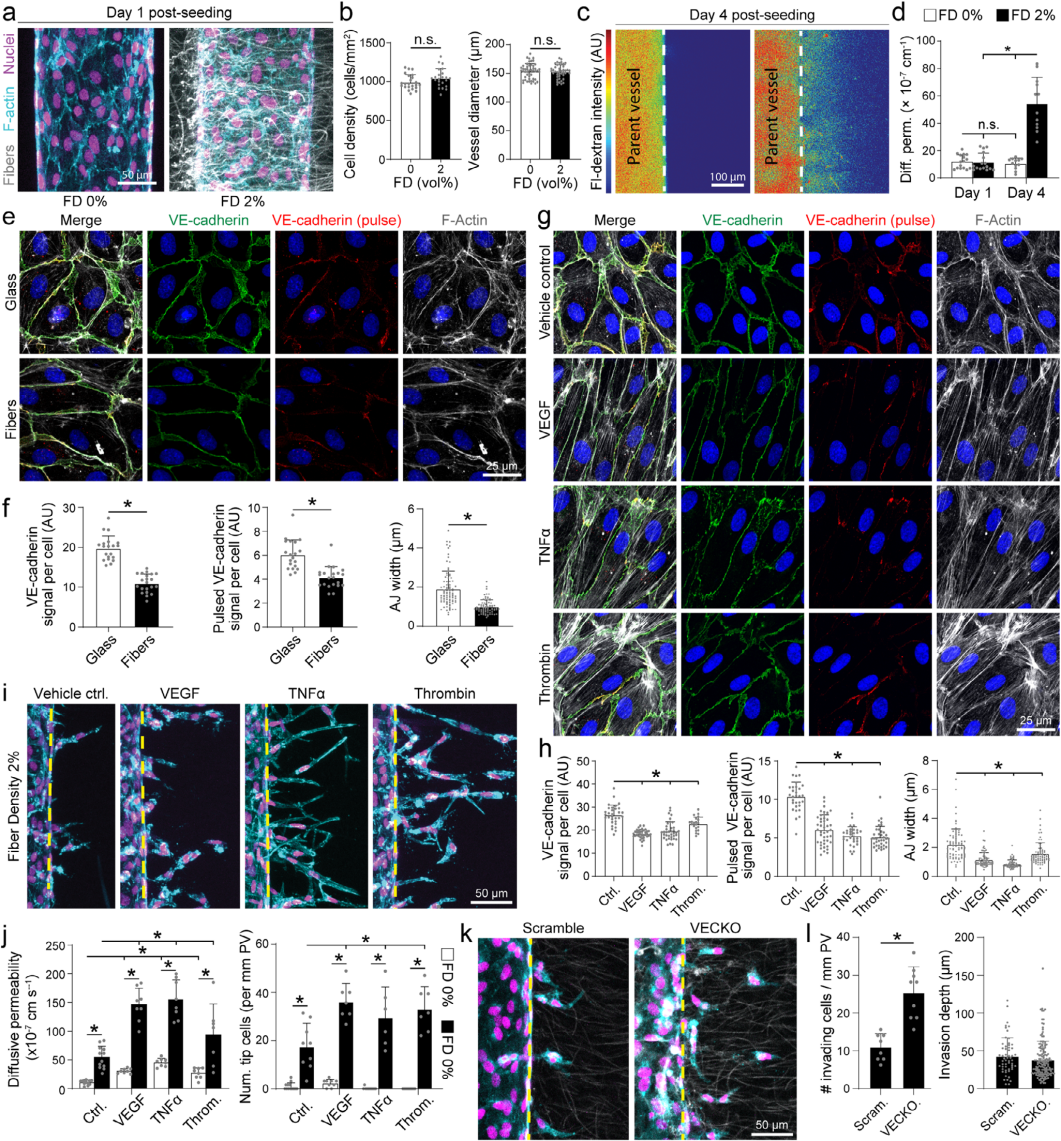
VE‐cadherin destabilization increases vessel permeability and ATEC activation. (a,b) Representative maximum intensity projection images of microvessels with or without FD 2% cell‐adhesive fibers after one day of culture (a) along with corresponding quantification of vessel wall cell density and microvessel diameter (b). Data representative of N=4 replicate studies with n>24 fields of view analyzed per condition. (c) Representative single z‐slice (at mid‐plane of microvessel) following fluorescent dextran perfusion on day 4 of culture. (d) Quantification of vessel wall diffusive permeability of microvessels cultured for 1 or 4 days in FD 0% or 2% hydrogels. N = 4 biological replicates with n>12 fields of view analyzed per condition. (e,f) Representative images (e) of EC monolayers cultured for 2 days on glass coverslips or electrospun DexVS fiber matrices along with corresponding quantifications of VE‐cadherin signal per cell, pulsed VE‐cadherin signal per cell, and adherens junction (AJ) width as determined by segmentation of VE‐cadherin^+^ cell‐cell junctions (f). N=3 replicate studies with n>20 fields of view analyzed per condition for. Data representative of N=3 replicate studies with n>20 fields of view per condition. (g,h) Representative images (g) of EC monolayers cultured for 2 days on fibrous matrices treated with vehicle (PBS), VEGF (50 ng/ml), TNFα (50 ng/ml), or thrombin (2 U/ml) along with corresponding quantifications (h) of VE‐cadherin signal per cell, pulsed VE‐cadherin signal per cell, and adherens junction (AJ) width as determined by segmentation of VE‐cadherin^+^ cell‐cell junctions. Data representative of N=3 replicate studies with n>25 fields of view analyzed per condition. (i,j) Representative maximum intensity projection images (i) of microvessels with FD 2% cell‐adhesive fibers treated with vehicle (PBS), VEGF (50 ng/ml), TNFα (50 ng/ml), or thrombin (2 U/ml) after 4 days of culture along with quantification (j) of vessel wall diffusive permeability and number of invading ECs. Data representative of N=4 replicate studies with n>6 fields of view analyzed per condition. Yellow dashed lines indicate parent vessel edge. (k–m) Western blot showing successful VECAD‐knock out (k) and representative maximum intensity projection images (l) of microvessels with FD 2% cell‐adhesive fibers composed of scramble control of VE‐cadherin KO ECs after 4 days of culture along with corresponding quantification (m) of the number of invading ECs and their invasion depth. Data representative of N=3 replicate studies with n>8 fields of view analyzed per condition number of invading cells and n>50 cells for invasion depth. All experiments presented were conducted with HUVECs. All data presented as means ± standard deviations; * indicates a statistically significant comparison with *p*<0.05 (b, c, f, l: student's t‐test, h: one‐way analysis of variance with Tukey's post hoc test, j: two‐way analysis of variance with Tukey's post hoc test).

Taken together, our results demonstrate that ECs within arteriole/venule‐scale vessels sense heightened perivascular fiber density, which promotes EndMT signaling to drive ATEC formation from an otherwise quiescent endothelium. Although microvessel morphology, cell density, and barrier function were all equivalent on day 1, three additional days of culture led to a 5‐fold increase in permeability for vessels exposed to a heightened perivascular fiber density (FD 2%) as compared to controls (FD 0%), which retained low permeability throughout the duration of study (Figure 4a–d). As increased vessel permeability and EndMT signaling have both been associated with the destabilization of VE‐cadherin‐based AJs [9, 59] we next examined whether EC adhesion to a fibrous topography directly modulated AJs.

We assessed AJs via VE‐cadherin immunostaining in confluent EC monolayers cultured on ‘flat’ substrates (lacking fibrous topography) as compared to ∼2D substrates composed of identical fibers deposited on glass coverslips [51, 60]. In line with our observations of decreased barrier function as determined by microvessel permeability measurements, EC monolayers on fibrous substrates were characterized by decreased overall VE‐cadherin intensity at AJs and VE‐cadherin‐containing AJ width (Figure 4e,f). We next examined relative VE‐cadherin stability in response to fibrous topography by live pulse‐labeling with a non‐perturbing, fluorescently tagged anti‐VE‐cadherin antibody that binds to the extracellular domain of VE‐cadherin. Relatively higher intensity of pulse‐labeled VE‐cadherin would be indicative of slower VE‐cadherin turnover [61]. EC monolayers on a fibrous topography possessed lower levels of pulsed anti‐VE‐cadherin antibody intensity at AJs as compared to non‐fibrous ‘flat’ controls (Figure 4e,f), suggesting that decreased junctional VE‐cadherin observed in EC monolayers on fibrous topography may be due in part to increased turnover rates at which VE‐cadherin is internalized from AJs.

VE‐cadherin abundance at AJs appeared to be modulated by EC interactions with a fibrous topography, and previous studies have implicated AJ stability vs. disassembly to be critical determinants of EC quiescence vs. activation. To test whether destabilization of AJs promotes fiber‐induced ATEC formation, we next examined ATEC formation after treatment of parent vessels with three well‐established vessel permeability agonists ‐ VEGF, TNFα, and thrombin, all of which have been implicated in the lung injury response [62, 63, 64]. We first confirmed that previously reported concentrations of VEGF, TNFα, or thrombin decreased VE‐cadherin AJ localization, AJ width, and pulsed VE‐cadherin signal intensity when delivered to EC monolayers cultured on ‘flat’ substrates relative to vehicle control (Figure 4g,h). As expected, applying the same concentrations of the three permeability agonists to microvessels in nonfibrous control hydrogels (FD 0%) resulted in modest but significant increases in vessel wall permeability, however EC invasion did not occur and microvessels remained morphologically identical to controls (Figure 4i,j and Figure S6). In contrast, microvessels with heightened perivascular fiber density (FD 2%) in combination with any of the three permeability agonists resulted in significantly higher permeability values relative to vehicle or nonfibrous control microvessels (Figure 4i,j). Concurrent with these decreases in barrier function, treatment with all three molecules led to increases in ATEC formation as quantified by invading ECs above levels of vehicle controls. To directly test the role of VE‐cadherin in gating fiber‐induced ATEC formation, we examined an extreme scenario of ECs completely lacking VE‐cadherin by complete knockout of VE‐cadherin (VE‐cadherin knockout, VECKO) via CRISPR‐Cas9 editing (Figure S7). Microvessels formed from VECKO ECs in matrices with heightened fiber density revealed heightened EC invasion as compared to scrambled gRNA controls (Figure 4k,l). Collectively, these results demonstrate that EC adhesion to matrix fibers destabilizes VE‐cadherin at AJs, thereby enabling ATEC activation and invasion, an effect further exacerbated by lung injury–associated mediators such as VEGF, TNFα, and thrombin.

### 2.5 Transcriptomic Analysis of ATECs Reveals EndMT Signaling and a Proinflammatory Phenotype

**FIGURE 5:**
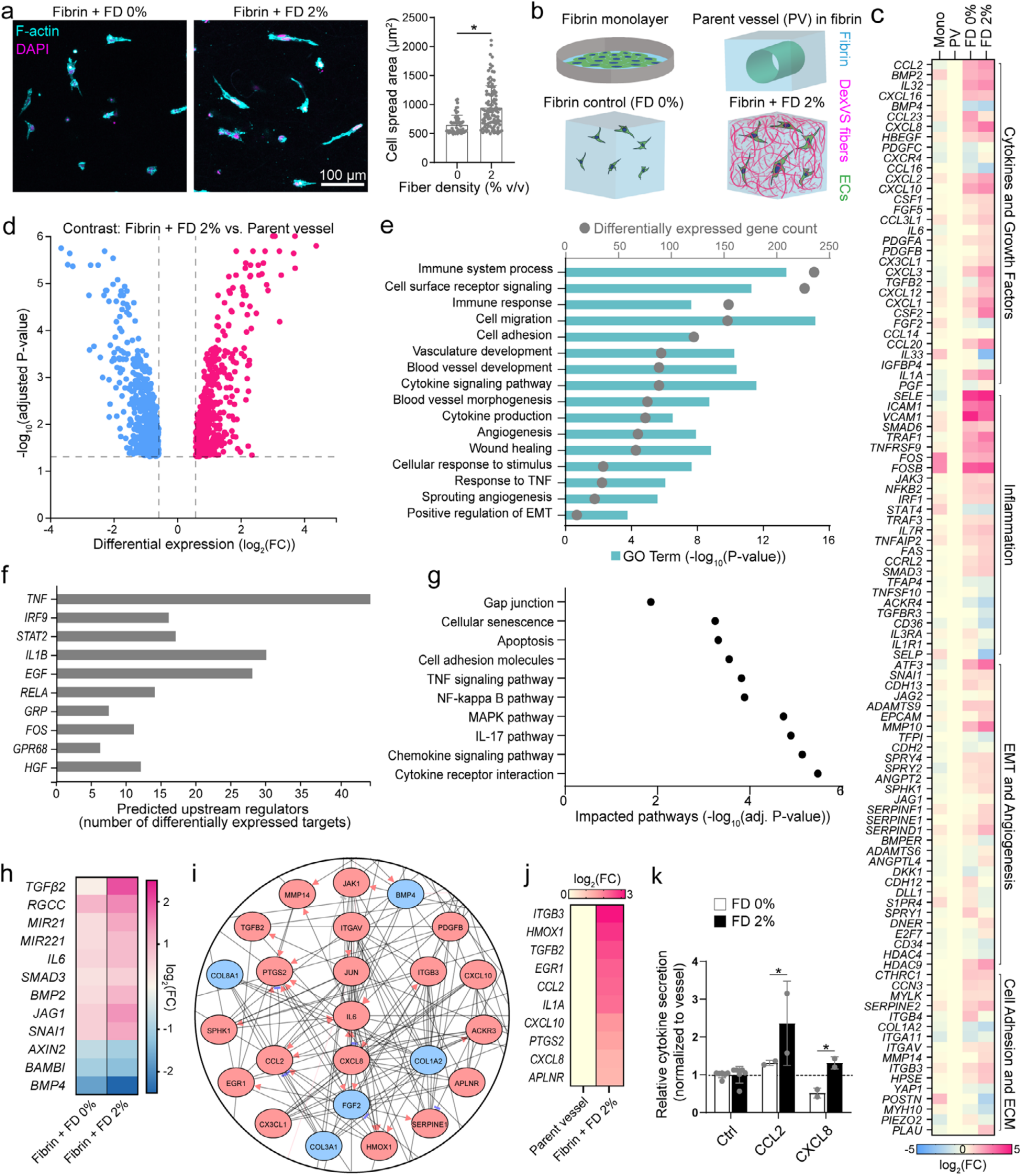
Transcriptomic analysis of ATEC suggests an inflammatory and pro‐fibrotic secretory phenotype. (a) Representative maximum intensity projection images of HUVECs embedded within control fibrin (FD 0%) or fibrous (fibrin + FD 2%) hydrogels cultured over 3 days to model ATECs at sufficient numbers for transcriptomic analysis. Corresponding quantification of EC spread area. Images and quantification representative of N=3 replicate experiments. (b) Schematic depiction of conditions for transcriptomic analysis. Experiment was performed once with n>2 technical replicates. (c) Expression of several curated gene categories relative to parent microvessels after 3 days of culture. (d) Volcano plot visualizing differentially expressed genes of HUVECs in fibrin + FD 2% hydrogels compared to parent microvessel HUVECs after 3 days of culture. (e) *p*‐value and number of differentially regulated genes for Gene Ontology (GO) terms comparing HUVECs in Fibrin + FD 2% to parent microvessel HUVECs. (f) Top 10 genes due based on differentially expressed genes predicted to be upstream regulators of these identified genes. (g) Top 10 differentially regulated GO terms based on impacted pathways. (h) Differentially expressed genes within GO term “positive regulation of EMT. (i) Predicted interactome for differentially expressed genes relevant to fibrosis. (j) Curated genes of interest differentially expressed by HUVECs in fibrin + FD 2% relative to parent microvessel HUVECs. (k) Cytokine antibody membrane array detection of CCL2 (MCP1) and IL‐8 (CXCL8). Experiment was performed once with n=2 technical replicates. All experiments were conducted with HUVECs. All data presented as means ± standard deviations; * indicates statistically significant comparison with *p*<0.05 (a: student's t‐test. k: two‐way analysis of variance with Tukey's post hoc test).

We next explored the EC transcriptome as a function of EC organization (i.e. multicellular microvessels vs. isolated cells) and engagement with a 3D fibrous topography with the goal of characterizing differential gene expression and biological processes characteristic of ATECs. For multicellular samples, mRNA was harvested from pooled MPS microvessels cultured for 3 days and normalized to a control consisting of a confluent monolayer cultured on fibrin (Figure 5a–c). Given the limited number of ATECs formed per MPS microvessel (∼50) and our inability to isolate them from ECs within microvessels, we modeled ATECs (i.e. isolated ECs adhering to matrix fibers) at sufficient quantities by embedding ECs in fibrous (FD 2% v/v) fibrin hydrogels (ATEC model) and included a non‐fibrous (FD 0% v/v) condition with the goal of segregating the effects of 3D encapsulation from those arising from cell adhesion to matrix fibers. Encapsulated ECs cultured for 3 days (prior to harvest for transcriptomic analysis) in FD 2% matrices possessed greater projected spread areas and longer protrusions compared to non‐fibrous controls (Figure 5a), in line with our prior studies demonstrating that cell‐adhesive fibers promote EC spreading [65, 66]. Although ECs in this condition lacked a shared orientation due to the random orientation of fibers in bulk‐casted gels, individually these cells adopted a uniaxial, spindle morphology and extended protrusions along fibers, identical to invading ATECs in our MPS studies (Figure 2).

Comparing confluent 2D EC monolayers adhering to fibrin to ECs isolated from MPS microvessels, relatively few differentially regulated genes were identified (Figure S8a), indicating comparable gene expression programs despite fluidic perfusion in MPS cultures and minor differences in substrate curvature. We next focused our analysis on whether single ECs engaging a matrix with heightened fiber density adopted a transcriptomic signature that might promote fibrosis‐associated signaling as compared to quiescent ECs within multicellular microvessels.

ECs cultured in 3D in FD 0% and 2% fibrin hydrogels possessed 294 and 1071 differentially expressed genes, respectively as compared to EC monolayers (Figure S8b,c). Heightened fiber density (FD 2% v/v), which gave rise to ATECs in earlier MPS experiments (Figures 2, 3, 4), significantly changed Gene Ontology (GO) terms associated with cell migration, cell adhesion, angiogenesis, and positive regulation of EMT, all of which involved genes implicated in tip cell activation, migration, or function (Figure 5e). Supporting our earlier observations of increased SNAI1 immunostaining in ATECs (Figure 3c) and gene expression resulting from EC adhesion to a fibrous topography (Figure S5), *SNAI1* gene expression increased along with several other EndMT‐associated genes such as *MMP14* and multiple TGF‐β‐associated genes (*TGFB2, BMP2, SMAD3*) (Figure 5c). Expression of *CTHRC1*, recently established as a unique marker of disease‐associated myofibroblasts in the heart and lung, was comparably increased in both FD 0% and FD2%, although we noted decreased expression of genes associated with matrix secretion and remodeling (*COL1A2* and *POSTN)*, two well‐accepted markers of myofibroblasts, suggesting that although ECs may undergo EndMT, transition to bona fide myofibroblast may require additional soluble and/or physical factors not present in these studies [7, 10, 67]. Consistent with these findings, only a sub‐population of lineage‐traced ECs were also αSMA^+^, and αSMA within these cells did not localize to stress fibers but instead appeared uniformly diffuse throughout the cytosol (Figure 1).

Pathway analysis based on differentially expressed upstream predicted to regulate these genes identified several inflammatory cytokines and growth factors including TNFα, IL1β, EGF, and HGF (Figure 5f). Cytokine, chemokine, and inflammatory signaling, gap junctions and cell adhesion, senescence, and apoptosis were among the top 10 impacted pathways. Examining the GO term “positive regulation of EMT” revealed heightened expression of the majority of genes within fibrin + FD 2% hydrogels as compared to control hydrogels (FD 0%), with TGF‐β2 expression uniquely upregulated in modeled ATECs (Figure 5h). The predicted interactome for differentially expressed genes relevant to fibrosis indicated positive feedback between growth factors (*TGFβ2, PDGFB*), inflammatory cytokines and chemoattractants (*IL6, CCL2, CXCL8, and CX3CL1*), and integrins (*ITGAV, ITGB3*), with significantly heightened expression by modeled ATECs compared to parent microvessel HUVECs (Figure 5i,j).

Furthermore, we utilized a cytokine antibody membrane array to detect whether other secreted cytokines were elevated in response to heightened fiber density. An inflammatory cytokine immunoblot array indicated elevated levels of MCP1 (CCL2) and IL8 (CXCL8) secreted by modeled ATECs compared to parent vessel ECs (Figure 5k and Figure S9). In line with these findings, both CCL2 and CXCL8 were significantly upregulated at the transcriptomic level (Figure 5c). Taken together, our data demonstrates ATECs express genes associated with EndMT markers and angiogenesis, as well as pro‐inflammatory and pro‐fibrotic cytokines suggestive of a disease propagating phenotype that is distinct from EndMT‐derived myofibroblasts.

### 2.6 A VE‐Cadherin–TGF‐βR2 Interaction Gates Fiber‐Induced TGF‐β Signaling in ECs

**FIGURE 6:**
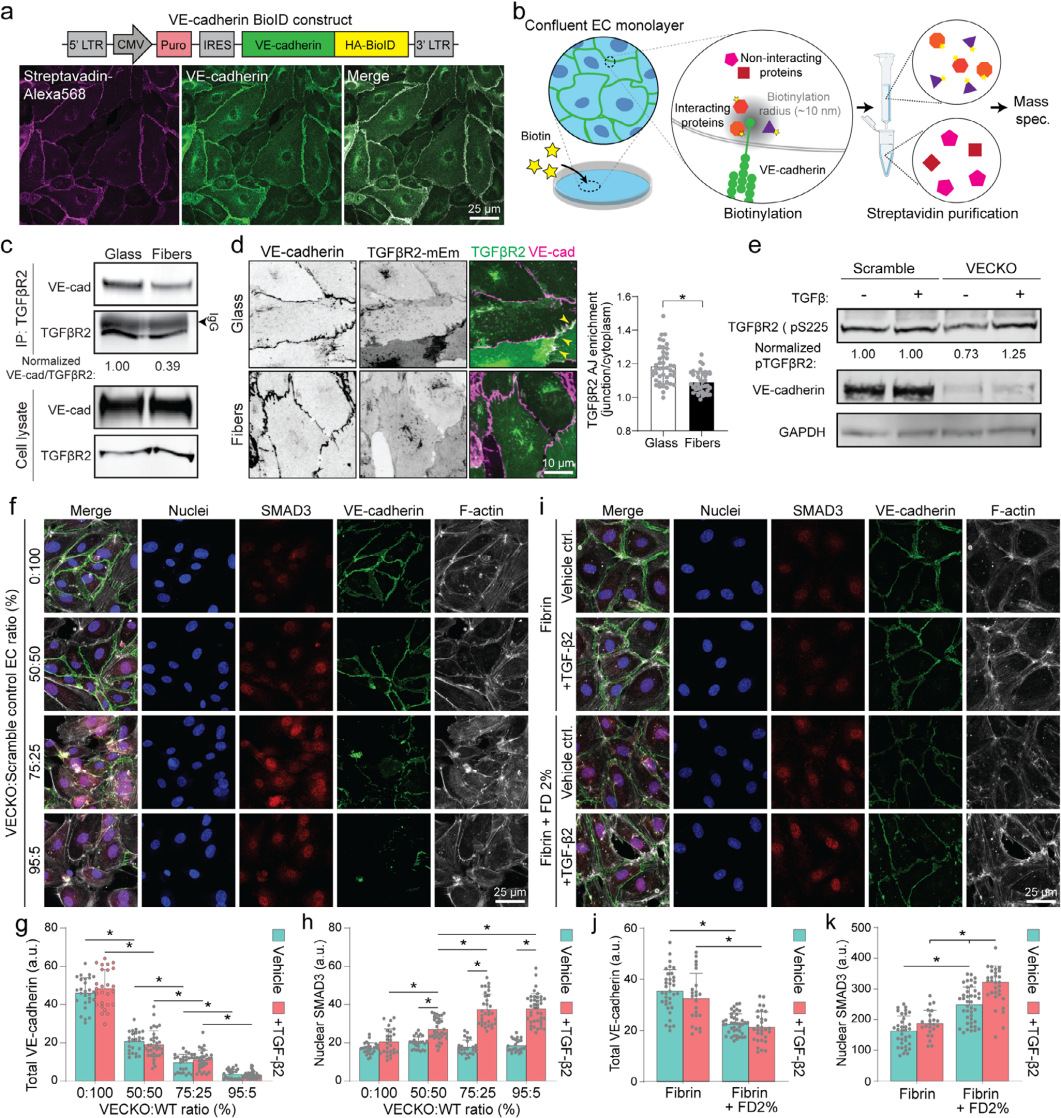
EC engagement to fibrous topography modulates TGF‐β signaling via VE‐cadherin–TGF‐βR2 dependent interactions. (a) Diagram depicting VE‐cadherin BioID construct. hMVECs stably expressing VE‐cadherin‐BioID were incubated in media containing biotin and then stained for VE‐cadherin and biotinylated proteins (Streptavidin‐AF568). (b) Schematic of VE‐cadherin‐BioID mass spectrometry screen to identify proteins interacting with VE‐cadherin. (c) Western blot micrograph of TGF‐βR2/VE‐cadherin co‐immunoprecipitation from ECs cultured on flat glass or DexVS‐RGD fibrous substrates. Blot representative of N=3 replicate experiments. (d) Representative images of hMVECs expressing TGF‐βR2‐mEmerald cultured on flat substrates or fibrous topographies immunostained for VE‐cadherin along with quantification TGF‐βR2 localization to VE‐cadherin AJs. (e) Western blot of scrambled control or VECKO HUVEC lysates treated with or without TGF‐β2 ligand (10 ng/mL). Blot representative of N=3 replicate experiments. (f–h) Representative images (f) and quantifications of total VE‐cadherin intensity (g) and nuclear SMAD3 (h) as a function of varied ratios of scrambled control HUVECs and VECKO HUVECs. (i–k) Representative images (i) and quantifications of total VE‐cadherin intensity (j) and nuclear SMAD3 (k) as a function of HUVEC adhesion to pure fibrin (FD 0%) or fibrin + FD 2%. Images and quantification representative of N=3 replicate studies each with n>6 field of view analyzed per condition. All data presented as means ± standard deviations; * indicates statistically significant comparison with *p*<0.05 (d: two‐sided student's t‐test. g‐h: two‐way analysis of variance with Tukey's post hoc test).

Our data suggest that EC adhesion to a fibrous topography destabilizes AJs, thereby enabling EndMT signaling and ATEC formation (Figure 4), and that ATECs express TGF‐β2 which has been shown to drive EndMT (Figure 5). As AJ and VE‐cadherin turnover have previously been shown to regulate EC behavior in response to growth factors such as VEGF [68], we next sought to identify potential molecular mechanisms by which VE‐cadherin stability at AJs might regulate TGF‐β signaling in ECs. To do so, we employed proximity ligation mass spectrometry (BioID) to profile proteins that associate with VE‐cadherin in quiescent, confluent monolayers reflecting the adhesive state of ECs within quiescent microvessels (Figure 6a,b), supported by the above finding that EC monolayers on fibrin and in MPS microvessels are transcriptionally similar. In quiescent 2D EC monolayers, analysis of the most abundant proteins identified via mass spectrometry expectedly revealed prominent associations with core AJ plaque proteins including α, β, γ, and δ‐catenins. However, among the most abundant (top 15) VE‐cadherin interactors, we unexpectedly identified a prominent association between VE‐cadherin and the receptor tyrosine kinase transforming growth factor β receptor 2 (TGF‐βR2) (Figure S10). As our transcriptomic and functional analyses associate EndMT signaling with ATECs, and TGF‐β signaling has been established to drive EndMT, we next investigated whether this putative VE‐cadherin‐TGF‐βR2 interaction is modulated by EC engagement to a fibrous topography.

To test whether TGF‐βR2 and VE‐cadherin association is influenced by EC engagement to matrix fibers, EC monolayers were cultured on ‘flat’ glass substrates or substrates with fibrous topography, lysed, and VE‐cadherin interactors were analyzed by co‐immunoprecipitation. We confirmed that VE‐cadherin co‐immunoprecipitates with TGF‐βR2 and that less TGF‐βR2 associates with VE‐cadherin in ECs cultured on fibers compared to those cultured on flat substrates (Figure 6c), suggesting that EC engagement to a fibrous topography attenuates this VE‐cadherin–TGF‐βR2 interaction. Corroborating this finding, expression of an mEmerald‐fusion tagged TGF‐βR2 in ECs on flat vs. fibrous topographies showed decreased enrichment of TGF‐βR2 at AJs in ECs engaging a fibrous topography (Figure 6d). To directly test whether VE‐cadherin interactions with TGF‐βR2 modulated the EC response to TGF‐β ligands independent of topography, we next assayed TGF‐βR2 phosphorylation [69] in scrambled control vs. VECKO EC monolayers on glass substrates (Figure 6e). Stimulation with TGF‐β ligand did not alter TGFβ‐R2 phosphorylation in control EC monolayers. In VECKO EC monolayers, basal TGF‐βR2 phosphorylation was lower than control ECs; however, in contrast, TGF‐βR2 phosphorylation markedly increased in response to stimulation with TGF‐β (Figure 6e). Taken together, we identify a VE‐cadherinTGF‐βR2 interaction that is disrupted upon EC engagement with a fibrous matrix, thereby permitting increased TGF‐βR2 phosphorylation in response to TGF‐β ligands.

To test whether TGF‐β signaling is modulated by VE‐cadherin and AJ stability, we next performed mosaic studies with scramble control and VECKO ECs at varying ratios to modulate VE‐cadherin localization to AJs while maintaining a constant cell density. To assess TGF‐β signaling, we treated cells with TGF‐β2, as our prior findings demonstrated that ATECs express this TGF‐β ligand, and quantified nuclear SMAD3 localization, as TGF‐β2 binding to TGF‐β receptors leads to downstream nuclear translocation of SMAD2/3/4 transcription factor complexes [70]. Indeed, increased ratios of VECKO:control ECs resulted in decreased VE‐cadherin intensity at AJs and corresponding increases in TGF‐β2 induced SMAD3 nuclear localization (Figure 6f–h). Additionally, we employed a scratch wound assay to vary AJs within the same samples where ECs at the scratch margin should possess less AJs than those fully surrounded by neighboring cells. At the scratch wound margin, ECs possessed decreased VE‐cadherin and increased nuclear SMAD3 compared to those in regions distal to the scratch with increased VE‐cadherin and decreased nuclear SMAD3 (Figure S11a–c). Directly modulating cell‐cell adhesion and AJ assembly by tuning EC seeding density similarly resulted in increased nuclear SMAD3 with lower cell density and resulting AJ formation (Figure S11d–f). Lastly, we compared control (FD 0%) with fibrous (FD 2%) matrices and found that fibrous topography in the absence of TGF‐β2 led to increased nuclear SMAD3, which was further increased in the presence of TGF‐β2 (Figure 6i–k). Altogether, these studies demonstrate that VE‐cadherin at AJs inhibits downstream TGF‐β‐signaling (as evidenced by decreased nuclear SMAD3 expression) via an interaction with TGFβ‐R2, and that this interaction is disrupted upon VE‐cadherin destabilization when ECs engage fibrous ECM.

### 2.7 TGF‐β2‐Induced EC Apoptosis May Underlie Fibrosis‐Associated Microvascular Rarefaction

**FIGURE 7:**
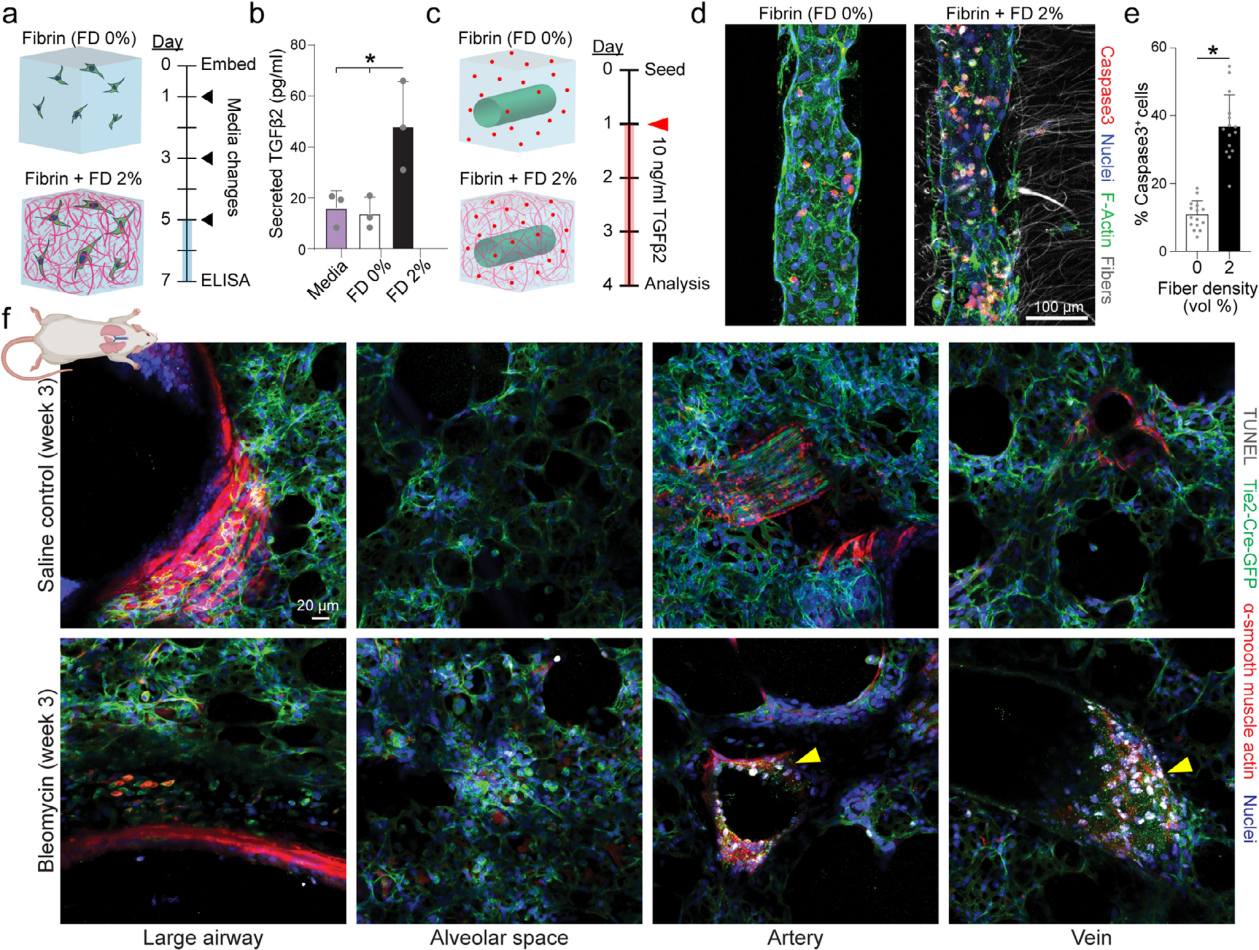
EC engagement to a matrix with heightened fiber density potentiates apoptosis in vitro and in vivo. (a) Schematic of study conditions and timeline for assessing TGF‐β2 secretion from ECs encapsulated in fibrin with (FD 2%) or without (FD 0%) heightened fiber density. (b) Quantification of TGF‐β2 secretion in culture media was performed via microfluidic ELISA. Data presented is from a single experiment with n=3 biological replicates. (c) Schematic of study conditions and timeline to assess the impact of heightened perivascular fiber density on TGF‐β2‐mediated apoptosis. (d) Representative images of parent vessel EC apoptosis in response to TGF‐β2 (10 ng/ml) via caspase3 immunostaining and corresponding quantification (e). (f) Representative images of large airways, alveolar space, arterioles, and venules from saline control and bleomycin treated mouse lungs at 3 weeks with TUNEL^+^ apoptotic ECs shown in white.

Given the implication of EC TGF‐β2 secretion in the above studies (Figures 5 and 6) and the broad role of TGF‐β growth factors as potent mediators of fibrosis via influencing fibroblast‐myofibroblast activation and EndMT, we employed a microfluidic ELISA assay to quantitatively measure protein levels as a function of EC engagement to matrix fibers. TGF‐β2 secretion from ECs encapsulated in matrices with heightened fiber density was 3–4 fold higher than nonfibrous controls, which were comparable to baseline levels of TGF‐β2 within the serum component of EGM2 (Figure 7a,b).

Together, our transcriptomic and secreted factor analyses implicate ATECs as a potential fibrosis‐propagating EC phenotype via inflammatory cytokine and TGF‐β2 secretion, which may drive fibrosis through action on other tissue resident cells (i.e. macrophages, fibroblasts, parental endothelium). Focusing on the latter possibility, we tested whether ATEC‐secreted TGF‐β2 may influence arteriole/venule‐scale parent vasculature, by exposing parent microvessels in control (FD 0%) and fibrous (FD 2%) hydrogels to exogenous TGF‐β2 (Figure 7c). TGF‐β2 induced apoptosis to a greater degree in microvessels with heightened perivascular fiber density as determined by positive immunostaining for cleaved‐caspase‐3 [71] (Figure 7d,e). Additionally, blocking TGF‐βRI (Alk5) via LY2109761 in microvessels with heightened perivascular fiber density (FD 2%) significantly reduced apoptosis and intriguingly led to more ATEC invasion (Figure S12), adding further support for a relationship between TGF‐β2 and apoptosis of parent microvessel‐resident ECs. In line with these in vitro findings, TUNEL staining confirmed evident EC apoptosis in bronchial arterioles and venules of bleomycin‐dosed lungs 3 weeks after injury, in stark contrast to saline‐treated controls (Figure 7f). Cells resident to airways or alveolar regions demonstrated no indication of apoptosis, although abundant dual positive GFP/αSMA ECs were present.

A common observation across multiple animal models of lung fibrosis is microvasculature rarefaction toward the late‐stage of the disease, during which both fiber density and TGF‐β levels increase [72, 73, 74]. Our data demonstrates that ECs engaging a fibrous topography destabilize VE‐cadherin which renders ECs more susceptible to TGF‐β ligands and downstream signaling. The finding that VE‐cadherin localization to AJs confers protective shielding from TGF‐β‐induced apoptosis may offer a promising therapeutic approach to prevent microvascular injury, rarefaction, and resulting tissue hypoxia in fibrotic diseases.

## 3 Discussion

**FIGURE 8:**
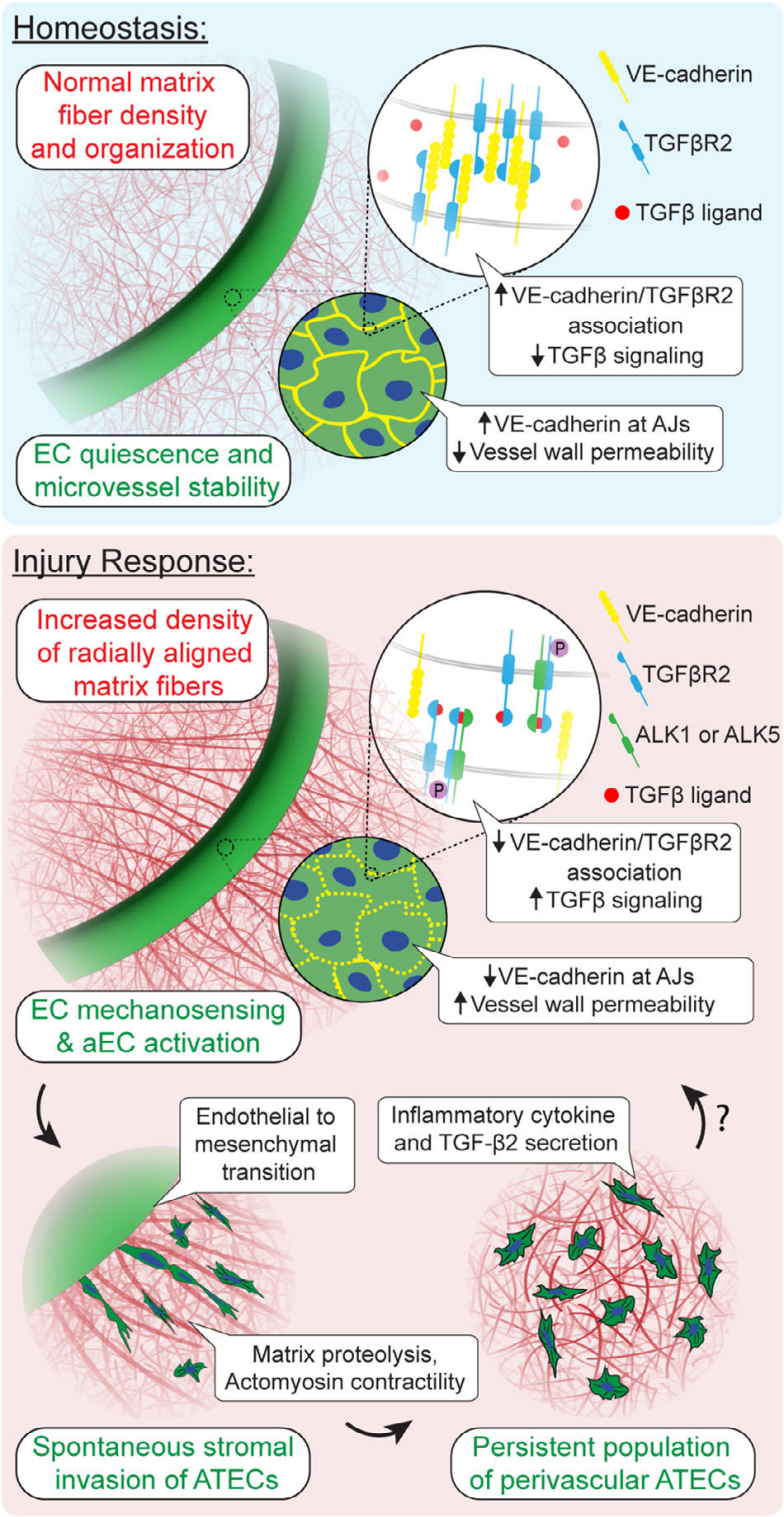
Summary schematic of key findings and outstanding questions (denoted with question mark).

While numerous soluble factors have been identified as activating signals for quiescent ECs and mediators of tip cell invasion [75], the role of physical cues presented by the ECM in directly driving this process or synergizing with soluble cues is understudied. EC activation and changes in ECM properties, such as fibrillar matrix density and organization, have both been observed following wound healing and during pathologic processes such as fibrosis. Here, we demonstrate how perivascular ECM fiber density directly influences ECs of arteriole/venule‐scale endothelium. Using an MPS platform of microvessels embedded within a tunable fibrous hydrogel composite, we found that YAP‐mediated mechanosensing of perivascular fiber density results in ATEC activation and invasion in part mediated by EndMT signaling. ATECs were often individualized cells and did not form lumenized capillaries, similar to those observed in our in vivo EC‐lineage tracing studies. Mechanistically, elevated fiber density destabilizes VE‐cadherin at AJs, diminishes vessel barrier function, and enhances TGF‐β signaling via disruption of a previously unreported VE‐cadherin–TGF‐βR2 interaction. Furthermore, ATECs engaging matrix with heightened fiber density possessed transcriptomic profiles and secreted factors suggestive of a pro‐inflammatory and pro‐fibrotic state. Altogether, our work identifies how enhanced perivascular fiber density observed during fibrogenesis regulates EC phenotype to generate ATECs which may play unappreciated roles in reinforcing fibrotic cascades (Figure 8).

Given that ECM remodeling in response to bleomycin and in human idiopathic pulmonary fibrosis is highly heterogeneous across the lung, we employed fibrous hydrogel composites integrated with a microvessel MPS to better control the local microenvironment and interrogate underlying pathophysiology. Using this approach, we found that adhesion of microvessel ECs to surrounding fibers led to decreased vessel barrier function and junctional weakening through increased turnover of VE‐cadherin at AJs. These observations raise the question of whether endothelial mechanosensing of discrete fibers is similar to, or distinct from, that induced by bulk matrix stiffening. Notably, increasing bulk matrix stiffness (e.g. via density or crosslinking) would restrict cell invasion, in contrast to the permissive environment provided by fibrous hydrogel composites. Cell adhesion to ECM fibers in other contexts has recently been demonstrated to result in focal adhesions with distinct integrin and focal adhesion compositions [76], which may underlie our observed differences in ECs plated on flat vs. fibrous substrates presenting the same adhesive ligand. Modeling increased subendothelial stiffness using hydrogels with tunable stiffness has been reported to increase EC TGF‐β2 secretion [77], which we also observe in response to heightened fiber density. Therefore, it is plausible that both conventional means and more physiologic strategies to modulate matrix stiffness, as explored here, can drive similar downstream gene regulatory network changes.

Fibrotic tissues have been characterized by microvascular abnormalities, but the relationship between angiogenesis and fibrogenesis remains unclear. Many studies report an increased angiogenic response based on increases in EC numbers (e.g. CD31^+^ cells in a histological section) within a given injured or fibrotic tissue, but careful analysis of the connectivity, perfusability, permeability, and therefore overall functional state of these newly formed EC structures has not been previously performed. Here, in a bleomycin‐induced lung injury model, we use a Tie2Cre‐mTmG lineage‐tracing murine model to show that ECs in the murine lung markedly expand by the peak of the bleomycin‐induced fibrotic response when α‐SMA^+^ stress‐fiber bearing myofibroblasts are most abundant. Coinciding with this expansion, we observed increased fibrillar ECM surrounding bronchial vasculature and the appearance of individualized, αSMA^+^ ATECs adjacent to bronchial arterioles and venules. Previous single cell RNA sequencing studies of tissues from interstitial lung disease patients identified a disease‐specific expansion of ECs, which were broadly termed peribronchial ECs [78, 79, 80]. Why ECs of large airway‐associated vasculature may be more susceptible to fibrosis‐associated activation remains unclear; however, one working model informed by our findings is rapid perivascular ECM remodeling following lung injury. Notably, bronchial venules possess thin walls with limited adventitia, and this lack of mural matrix support may increase EC sensitivity to changes in the perivascular matrix and/or more easily permit ATEC invasion into the perivascular space. Perhaps related to this, blood flow through the bronchial venous system is significantly (∼30%) reduced due to shunting of incoming blood flow to the pulmonary vasculature, which may further exacerbate this vulnerability [79, 81, 82].

Canonically, perivascular cells (PCs), including pericytes and smooth muscle cells, are critical mechanosensors and regulators of vascular stability and function. Recent work has refined this view, suggesting that PCs act in a matrix context‐dependent manner to regulate endothelial barrier function. In soft matrices, EC‐PC crosstalk modulated by NOTCH signaling stabilized VE‐cadherin at adherens junctions and reinforced the quiescent endothelial state [83]. In contrast, a matrix composed of stiff and bundled (larger) collagen fibers promoted a loss in barrier function, which was amplified when PCs were present but prevented by silencing NOTCH3 in PCs [84]. Stabilizing PC‐EC interactions are known to be disrupted during fibrogenesis [13]. A large body of evidence suggests that in pathological states, perivascular cells uncouple or dissociate from the endothelium and may differentiate into myofibroblasts that contribute directly to fibrotic matrix deposition [85, 86, 87]. Indeed, we observed a similar dissociation of MSCs (known to share transcriptional and functional similarities with perivascular cells including pericytes and smooth muscle cells [88, 89, 90, 91, 92]) in response to heightened perivascular fiber density, with ATEC formation proceeding similarly independent of the presence of mural cells (Figure S4). Microvessels composed of only ECs and a surrounding fibrous matrix therefore recapitulate the loss of perivascular support and allow us to directly interrogate the EC‐intrinsic mechanosensing response to newly deposited fibrillar ECM, without the confounding contributions of PC‐derived ECM remodeling, paracrine factors, and juxtacrine signaling that arise from co‐culture designs.

An intriguing observation was the non‐responsiveness of dermal MVECs to heightened fiber density, contrasting the robust ATEC formation seen in liver‐ and lung‐derived ECs. This discrepancy highlights the concept of EC heterogeneity and organ‐specificity [93]. The peribronchial ECs identified in vivo may be conditioned by the highly compliant mechanical environment of the airways, priming them to respond to pathological increases in perivascular matrix density. In contrast, dermal MVECs reside within a comparatively stiffer tissue environment and may therefore exhibit a more stable junctional phenotype or differential expression of key mechanosensors, thereby limiting ATEC formation. Thus, the EC‐intrinsic feed‐forward loop we present here may represent a pathological pathway with varied importance across organs. As such, further studies into the organ‐specific appearance and function of ATECs are needed.

In mice, EC activation by bleomycin‐induced injury occurs through YAP/TAZ signaling [78], and here we demonstrate that YAP inhibition via DMF blocks the formation of ATECs. YAP/TAZ signaling has been implicated in driving epithelial‐to‐mesenchymal transition and related transcriptional reprogramming in response to mechanical cues [27] which may extend to ECs. Consistent with this, previous studies in lung and other organs suggest a role for EndMT in fibrosis by directly contributing to the myofibroblast population [10, 19, 94]. In contrast, here we posit that fibrous ECM–induced EndMT promotes the formation of a disease‐mediating ATEC phenotype. Transcriptomic analysis, microfluidic ELISA, and cytokine profiling indicate that fibrous topography drives ECs toward a pro‐inflammatory, TGF‐β2–secreting phenotype. This is consistent with single‐cell RNA‐seq studies in bleomycin‐treated mouse lung, which identify expanded EC populations derived from bronchial venous ECs with specialized inflammatory functions [95]. It is therefore plausible that ATECs may play a key role in reinforcing fibrotic cascades through interactions with the neighboring microvasculature and other stromal cells including fibroblasts and macrophages. Supporting an EC‐centric feedback loop, exogenous addition of TGF‐β2 to simulate ATEC secretion resulted in elevated apoptosis in parent microvessels with heightened perivascular fiber density. As TGF‐β2 and fibrillar collagen density are both elevated during later phases of fibrosis [96], these studies may provide insight into the molecular mechanisms underlying the common observation of microvasculature rarefaction. Thus, rather than contributing to productive angiogenesis, ATECs may represent a maladaptive endothelial state that actively promotes fibrosis.

This work describes a novel VE‐cadherin‐gated mechanism controlling TGF‐β signaling dependent on AJ stability. Using BioID and mass spectrometry with confirmation via co‐immunoprecipitation experiments, we found that TGF‐βR2 directly interacts with VE‐cadherin, and that this interaction is modulated by EC adhesive state. In ECs engaging fibrous ECM, reduced association of TGF‐βR2 with VE‐cadherin coincided with VE‐cadherin destabilization at AJs and increased TGF‐β signaling, reflected by elevated TGF‐βR2 phosphorylation and downstream SMAD3 nuclear localization. In turn, EC adhesion to fibrous ECM destabilized VE‐cadherin from AJs and increased TGF‐β signaling as evidenced by elevated TGF‐βR2 phosphorylation and downstream SMAD3 nuclear localization. The precise mechanotransductive pathway linking fiber engagement to disruption of the VE‐cadherin/TGF‐βR2 complex remains to be defined. One possibility is that inside‐out signaling from fiber‐associated focal adhesions alters cytoskeletal tension at AJs, promoting VE‐cadherin turnover. Additionally, VE‐cadherin association with TGF‐βR2 may regulate receptor accessibility or heterodimerization, thereby modulating downstream signaling. Future work should also investigate the role of the canonical TGF‐β type I receptors ALK1 and ALK5, which compete for TGF‐βR2 binding and differentially regulate EC fate. Previous work in other contexts demonstrate that ALK5‐driven SMAD2/3 signaling (promoting EndMT, quiescence, or apoptosis) and ALK1‐driven SMAD1/5/8 signaling (promoting proliferation and migration) is critical for vascular homeostasis [97]. Our observations of both EndMT‐driven ATEC formation and parent vessel apoptosis suggest that the fiber‐induced, VE‐cadherin–gated mechanism we identify may disrupt this balance, potentially favoring the ALK5–SMAD2/3 axis. Together, these findings suggest that conditions that destabilize AJs ‐ such as engagement with fibrous ECM, inflammatory cues, or thrombin and VEGF signaling ‐ may potentiate TGF‐βR2‐dependent signaling, with broader implications for vascular dysfunction in fibrosis and inflammatory disease [98].

Lastly, it is also important to clarify that while ATECs studied in vitro share morphological characteristics with those observed in vivo, further studies are needed to assess transcriptomic or functional similarities. Our MPS model was intentionally reductionist to isolate the physical microenvironmental cues and phenotypic requirements for ATEC formation. This system, however, lacks the complex, multicellular signaling environment of a fibrotic lung, most notably the important contributions of immune cells. It is therefore highly likely that the ATECs in vivo possess a distinct and more complex phenotype, further modulated by paracrine interactions and reciprocal cross‐talk with immune cells. Our study suggests an EC‐intrinsic feedback loop where ATECs drive microvessel injury, but future work incorporating immune and other stromal cells will be essential to fully understand the effect of ATECs in heterocellular settings. Nonetheless, these studies provide new insights into how physical microenvironmental cues regulate EC phenotype, how pro‐inflammatory ATECs may contribute to the pathogenesis of fibrosis, and identify new mechanisms that may be targetable for treating pulmonary fibrosis and potentially other fibrotic diseases.

## 4 Materials and Methods

### 4.1 Reagents

All reagents were purchased from Sigma–Aldrich and used as received, unless otherwise stated.

### 4.2 Lung Injury Model in a Mouse Line Enabling EC Lineage Tracing

Male B6.Cg‐Tg(Tek‐cre)1Ywa/J transgenic mice were crossed with female Gt(ROSA)26Sortm4(ACTB‐tdTomato,‐EGFP)Luo mice to create Tie2cre/mTmG mice where all cells at baseline express membrane‐localized tdTomato, but upon Tie2‐driven Cre recombinase expression, flip expression to membrane‐localized GFP. 8–12 week‐old male mice were injected intratracheally with a single dose of 0.04 units bleomycin solubilized in PBS. Only male mice were used in this study as males exhibit a more severe response to bleomycin. Control mice lung were collected 3 weeks after dosing with PBS. Injured mice lungs and blood were harvested at week 3 after bleomycin instillation to examine the peak fibrotic response following lung injury. Warm PBS was perfused through the left ventricle at 5 ml/min until the liver turned white to clear lung tissue of red blood cells. Lungs were then inflated with 1.5 ml of 2% w/v low melting agarose (Sigma A9025) in 4% w/v PFA via a catheter inserted into the upper trachea. Upon agarose gelation, the entire lung was extracted and immediately fixed in 4% w/v PFA overnight at 4°C then washed with PBS three‐times the next day and stored in PBS with 0.01% sodium azide at 4°C until PCLS sectioning.

### 4.3 Lung Tissue Sectioning, Optical Clearing, Immunofluorescent Staining, and Imaging

The entire lung was first separated into left and right lobes then glued vertically on specimen holder to reflect original anatomical position. Lobes were then embedded in 2% w/v low melting agarose in PBS, to approximately match the stiffness of the tissue with the surrounding material during vibratome sectioning. PCLS (200 µm) were cleared using the advanced clear, unobstructed brain imaging cocktails (CUBIC) clearing method [37]. Specifically, lung sections were cleared in dH2O with 50% CUBIC‐L containing 10% w/w N‐butyldiethanolamine, and 10% w/w Triton X‐100 in dH2O for one day, and then 100% CUBIC‐L for another day. Following clearing, lung tissue was permeabilized for one day in PBS solution containing 20% v/v DMSO, 0.1% v/v tween‐20, 0.1% v/v triton X‐100, 0.02% v/v SDS, 0.1% w/v deoxycholate and 0.1% w/v tergitol NP40. Tissue sections were blocked and stored in PBS solution containing 0.2% v/v TritonX‐100, 6% v/v goat serum, and 10% v/v DMSO until staining.

Tissue was incubated with anti‐αSMA antibody (1:1000, Abcam #AB7817) or F4/80 antibody (1:250, Cytoskeleton #70076S) overnight in PBS containing 1 mg/ml heparin, 0.2% v/v tween‐20, 5% v/v DMSO, 3% v/v goat serum, and 1% w/v bovine serum albumin followed by a 12 h PBS wash overnight and overnight incubation in buffer containing Alexa‐conjugated secondary antibodies (1:1000, Invitrogen #A‐21133). Tissue was then stained by DAPI and NHS‐ester (1:3000, Invitrogen# A20006) sequentially, each for 30 min at room temperature with 3x PBS washes in between. Tissues were imaged in CUBIC‐R+M composed of 45% w/w antipyrine, 30% w/w N‐methylnicotinamide, and 0.5% v/v N‐butyldiethanolamine in dH2O via a laser‐scanning confocal microscope (Zeiss LSM800). TUNEL staining (Fisher, #C10619) following the manufacturer's instructions was utilized for visualizing DNA damage. Lung tissue stained by TUNEL did not go through CUBIC clearing due to chemical incompatibility and 25 µm Z‐stacks with 1 µm step size were taken using confocal at 20x only at the center of the tissue to avoid false positive signals at the plane of sectioning.

### 4.4 Organotypic Model Device Fabrication

Device moulds were designed in AutoCAD and printed via stereolithography (SLA) by Protolabs (Maple Plain, MN). Polydimethylsiloxane (PDMS, 1:10 crosslinker:base ratio) devices were replica‐casted from SLA printed moulds, cleaned with isopropyl alcohol and ethanol, and bonded to glass coverslips with a plasma etcher. Devices were treated with 0.01% (w/v) poly‐l‐lysine (PLL) and 0.5% (w/v) glutaraldehyde sequentially for 1 h each to covalently crosslink ECM to the walls of the device, thus preventing potential hydrogel compaction from cell‐generated forces. 160 µm stainless steel acupuncture needles (Lhasa OMS, Weymouth, MA) were dip‐coated with 1% w/v gelatin to facilitate removal without hydrogel fracture, inserted into each device, and sterilized by UV ozone. Hydrogel precursor solution was then injected into each device and polymerized around needles. Hydrogels were hydrated in EGM2 media at 37°C overnight (or greater than 12 h) to dissolve the gelatin layer and needles were extracted to form 3D hollow channels fully embedded within a crosslinked hydrogel and positioned 500 µm away from PDMS and glass boundaries. A chilled solution of 100 µg/ml matrigel diluted in PBS was perfused through channels and allowed to adsorb onto the hydrogel channel surface at 4°C overnight. Residual Matrigel was then rinsed with PBS twice.

### 4.5 Dextran Vinyl Sulfone Polymer Synthesis

Dextran (MW 86,000 Da, MP Biomedicals, Santa Ana, CA) was functionalized with vinyl sulfone groups as in [45, 46, 99]. Briefly, dextran (5 g) was dissolved in 0.1 m sodium hydroxide solution (250 mL) at room temperature. Divinyl sulfone (3.875 ml, ThermoFisher Scientific, Waltham, MA) was added and the reaction was carried out for 4 min with vigorous stirring (1500 RPM) at room temperature. The reaction was terminated by adjusting the pH to 5.0 with the addition of hydrochloric acid. The reaction product was dialyzed against milli‐Q water for 3 days with two water exchanges daily. The dialyzed reaction product was lyophilized for 3 days and characterized by ^1^H‐nuclear magnetic resonance spectroscopy in D_2_O. The degree of vinyl sulfone functionalization was calculated as the ratio of the proton integral (6.91 ppm) and the anomeric proton of the glucopyranosyl ring (5.166 and 4.923 ppm); a vinyl sulfone/dextran repeat unit ratio of 0.66 was determined.

### 4.6 Fiber Segment Fabrication

DexVS fiber segments were generated as in Matera et al. [45] DexVS was dissolved at 0.6 g ml^−1^ in a 1:1 mixture of Milli‐Q water and dimethylformamide with 0.015% Irgacure 2959 photoinitiator and 0.5 mm methacrylated rhodamine to enable visualization (Polysciences Inc., Warrington, PA). This polymer solution electrospun into fibers within an environment‐controlled glovebox held at 21°C and 30% relative humidity. Electrospinning was performed at a flow rate of 0.3 ml hour^−1^, gap distance of 5 cm, and voltage of −10.0 kV onto a grounded collecting surface attached to a linear actuator. Fiber mats were collected on glass slabs and primary cross‐linked under ultraviolet light (100 mW cm^−2^). After polymerization, fiber segments were resuspended in a 3 ml volume of PBS. The total volume of fibers was then calculated via a conservation of volume equation: total resulting solution volume = volume of fibers + volume of PBS. After calculating total fiber volume, solutions were re‐centrifuged, supernatant was removed, and fiber pellets were resuspended to create a 10% v/v fiber stock solution, which were then aliquoted and stored at 4°C. To enable cell adhesion, 2 mm arginylglycylaspartic acid (RGD, CGRGDS; GenScript, George Town, KY) was coupled to vinyl sulfone groups along the DexVS backbone via Michael‐type addition chemistry for 30 min, followed by quenching of excess VS groups in a 300 mm cysteine solution for 30 min.

### 4.7 Fibrin‐Fiber Composite Hydrogels

Fibrin hydrogels were prepared with fibrinogen from bovine plasma dissolved in PBS at 50 mg ml^−1^ stock concentrations. 10 mg ml^−1^ fibrinogen was prepared in PBS and crosslinked with thrombin (6 units per mg of fibrinogen) for 20 min at 37°C. For fibrous hydrogels, DexVS fiber segments were incorporated within the fibrin hydrogel precursor solution over a 0–2% v/v density. To generate fiber alignment perpendicular to the long axis of the parent vessel (0° alignment), fibrin precursor solution containing fibers were injected such that flow across acupuncture needles (rigid cylinder) generated a flow profile that aligned fibrils in the direction of flow. To generate fiber alignment parallel to the long axis of the parent vessel (90° alignment), fibrin precursor solution containing fibers were first injected into the device. Subsequent insertion of acupuncture needles generated flow profiles to align fibers in the direction of needle insertion. To generate collagen bundle‐embedded fibrin hydrogels, type I collagen rat tail (4.8 mg ml^−1^) was adjusted to a pH of 7.4 with sodium hydroxide and PBS on ice. Next, 80°C MQ water was added in a 1:1 ratio (2.4 mg ml^−1^ final concentration) and immediately vortexed for 30 s. Collagen bundles formed immediately during vortexing and were isolated via centrifugation (500 g for 4 min) and resuspended in fibrin hydrogels similarly to DexVS fibers.

### 4.8 Mechanical Testing

The Young's modulus of composite hydrogels were measured by atomic force microscopy (AFM; Nanosurf, Liestal, Switzerland) in contact mode. Indentations were made at a loading rate of 2 µm/s with silicon nitride cantilevers (AppNano, Mountain View, CA) with a nominal spring constant of 0.046 N/m and a 5 µm diameter spherical glass bead. Force‐displacement curves were taken at a minimum of 3 regions on each hydrogel and fit to the Hertz model assuming a Poisson's ratio of 0.5 to estimate the elastic modulus.

### 4.9 Microphysiological Model Seeding and Culture

Human umbilical vein, liver, lung, and dermal endothelial cells (Lonza, Switzerland) were cultured in endothelial growth media (EGM2, Lonza). HUVECs were passaged upon achieving confluency at a 1:4 ratio and used in studies from passages 4 to 9. Liver, lung, and dermal ECs were passaged at a 1:4 ratio and used in studies from passages 2 to 6. A 10 µl solution of suspended ECs (10 million cells ml^−1^ density) was added to one reservoir of the endothelial channel and inverted for 30 min to allow cell attachment to the top half of the channel, followed by a second round of seeding with the device upright for 30 min to allow cell attachment to the bottom half of the channel. ECs reached confluency and self‐assembled into stable parent vessels over 24 h. Media and chemokines were refreshed every 24 h and devices were cultured with continual reciprocating flow utilizing gravity‐driven flow on a seesaw rocker plate at 0.33 Hz. Tip cell formation in response to S1P gradients was assessed by adding 0–50 nM S1P diluted in EGM2 media added only to the chemokine channel for 3 days beginning 16 h after cell seeding. Fiber‐induced tip cell formation studies were cultured in EGM2 media alone for 3 days after 16 h post cell seeding. Agents for permeability agonist (VEGF121, TFNα, and thrombin) or pharmacologic (marimastat, blebbistatin, Y27632, dimethyl fumarate, and LY2109761) dosing studies were incorporated into EGM2 media and added to both the endothelial and chemokine channels for 3 days after 16 h post cell seeding. For studies examining the effect of supporting mural cells on EC activation, human mesenchymal stem cells (hMSCs, Lonza) were cultured in high‐glucose DMEM containing 5% fetal bovine serum and 1% P/S and were passaged upon achieving confluency at a 1:3 ratio and used from passages 3–6. For studies incorporating mural cells, hMSCs were first seeded into device channels as described above, at a 10 µl solution of 1.5 million cells ml^−1^. Endothelial cells were seeded as described above 1 h after hMSC seeding and attachment.

### 4.10 Diffusive Permeability Measurements

Diffusive permeability was quantified as in Polacheck et al. [100, 101] Briefly, fluorescent dextran (70 kDa Texas Red, Thermo Fisher) was dissolved in EGM2 media at 12.5 µg ml^−1^, perfused through endothelialized channels, and imaged at 1 s intervals to measure the flux of dextran across the endothelium into the ECM. The resulting diffusion profile was fit to a dynamic mass‐conservation equation as in [102] with the diffusive‐permeability coefficient (*P*_D_) defined by *J* = *P*_D_(*c*_vessel_ − *c*_ECM_), where *J* is the mass flux of dextran, *c*_vessel_ is the concentration of dextran in the vessel, and *c*_ECM_ is the concentration of dextran in the perivascular space.

### 4.11 VE‐Cadherin/TGF‐β Signaling Studies

For studies examining SMAD3 localization (Figure 6F–K and Figure S10), ECs were first treated with mitomycin C (20 µg ml^−1^ for 2 h) to inhibit cell proliferation and enable control over cell density throughout the duration of culture. For variable cell density SMAD3 studies (Figure S10d–f), ECs were seeded onto 10 mg ml^−1^ fibrin hydrogels adhered to 18 mm coverslips treated with PLL and glutaraldehyde at a low (25 K cells cm^−2^), medium (50 K cells cm^−2^), or high (100 K cells cm^−2^) cell density. The following day, samples were treated with 0 or 10 ng ml^−1^ TGF‐β2 (Peprotech) in EGM2 media (without serum or fibroblast growth factor (FGF), as FGF has previously been shown to inhibit TGF‐β signaling) and maintained for two days with media refreshed daily. For VECKO mosaic studies (Figure 6f–h), scrambled CRISPR control HUVECs and VECKO ECs (Figure S9) were seeded at varying ratios (0%, 50%, 75%, and 100% VECKOs) at a constant 100 K total cells cm^−2^ density onto 2D 10 mg ml^−1^ fibrin hydrogels. For 2D studies comparing fibrin vs. fibrin + DexVS‐RGD fibers (Figure 6i–k), hydrogel composites (“fibrin + fibers”) were generated by electrospinning DexVS fiber mats onto a dried gelatin‐coated coverslip. DexVS fiber mats were functionalized with RGD as in 3D studies. A solution of fibrin hydrogel was then added to the DexVS fiber mat and a second PLL/glutaraldehyde treated coverslip (as above) placed on top of the fibrin solution. After fibrin polymerization and hydration, the dried‐gelatin coverslip was removed, resulting in a DexVS‐RGD fibers localized to the top surface of the fibrin hydrogel. ECs were seeded on to fibrin control or fibrin + fiber substrates at a 100 K cells cm^−2^ density and dosed with TGF‐β2 as described above. For scratch wound studies (Figure S10a–c), glass coverslips were seeded with ECs at 100 K cells cm^−2^ density, physically ablated with a P1000 tip the following day, and dosed with TGF‐β2 identical as described, but with culture durations limited to 1 day, as at longer timepoints ECs were observed to completely close the scratched region.

### 4.12 Western Blotting

Samples for western blotting were collected from confluent EC monolayers cultured on glass coverslips with or without deposited electrospun DexVS‐RGD fiber [60] for 2 days after cell seeding and were treated with either EGM2 media, or EGM2 media containing 50 ng ml^−1^ VEGF121 (Peprotech, Cranbury, NJ), 50 ng ml^−1^ TNFα, or 2 U ml^−1^ Thrombin. To collect protein lysates, cells were collected with a cell scraper in chilled PBS solution and centrifuged to generate cell pellet. Supernatant was removed and replaced with RIPA buffer containing protease and phosphatase inhibitors. Following freeze‐fracture of cell membranes (10 min at −80°C), samples were centrifuged for 10 min at 20 000 g, and the supernatant was collected which contained the purified protein lysate. Protein lysate concentrations were measured using a BCA assay, and 30 µg ml^−1^ protein was loaded into a 4%–20% Tris‐Glycine Novex wedge well. Proteins were separated with electrophoresis for 90 min at 120 V in running buffer. Protein gels were then transferred to a PVDF membrane for 60 min at 20 V in transfer buffer at 4°C. PVDF membranes were then blocked in 5% w/v blocking grade milk buffer, and stained with an anti‐VE‐cadherin primary antibody (sc‐9989, 1:1,000, Santa Cruz Biotechnology, Dallas, TX) and β‐tubulin (66240‐1‐Ig, 1:10 000, Proteintech, Rosemont, IL) overnight on an orbital shaker at 4°C. Membranes were then rinsed in TBST and stained with horseradish peroxidase and imaged on a gel imager.

For TGFβR2 immunoprecipitations, samples were prepared as described in “VE‐cadherin/TGF‐β2 signaling studies” (above). Proteins were separated by SDS‐PAGE on 4%–12% Bis‐Tris gels and transferred to a nitrocellulose membrane at 4°C. Nitrocellulose membranes were blocked in 5% w/v dry milk powder dissolved in tris‐buffered saline with 0.1% Tween‐20 (TBST) for at least one hour. Membranes were incubated with mouse anti‐VE‐cadherin antibody (1:1000; sc‐9989, Santa Cruz Biotechnology, Dallas, TX) and rabbit anti‐TGFβR2 antibody (1:1000; PA535076, Invitrogen) dissolved in blocking buffer for at least two hours. Blots were washed three times with TBST following primary antibody incubation and were subsequently incubated with IRDye 800CW donkey anti‐rabbit IgG (1:10 000; LICOR) or IRDye 680RD donkey anti‐mouse IgG (1:10 000; LICOR) for at least one hour. Membranes were then rinsed at least three times with TBST and imaged using an Odyssey CLx LICOR Imaging System.

### 4.13 Transcriptomic Analysis

Samples for microarray analysis included 1) confluent EC monolayers cultured on 10 mg/ml fibrin hydrogel slabs, 2) confluent EC monolayers cultured in channels within 10 mg/ml fibrin hydrogels, 3) encapsulated, single ECs embedded within FD 0% 10 mg ml^−1^ fibrin hydrogels, and 4) encapsulated, single ECs embedded within FD 2% 10 mg ml^−1^ fibrin hydrogels. All conditions were cultured for 3 days upon which nattokinase (100 fibrinolytic units ml^−1^) was incorporated into EGM2 media for 15 min to digest the surrounding fibrin hydrogel to release attached ECs. ECs were pelleted and RNA isolation was performed via RNeasy mini kit per manufacturer's protocol. Purified RNA samples were submitted to the University of Michigan Sequencing Core for microarray analysis on an Affymetrix chip. Gene expression data was analyzed utilizing Advaita Bioinformatics software [103].

### 4.14 Cytokine and Growth Factor Secretion Analysis

To assess how fibrous topography influenced EC cytokine secretion, we employed a recently established microfluidic ELISA and an antibody cytokine detection array membrane. For both assays, samples were cultured for 7 days, with supernatant media refreshed every 2 days. Conditioned media collected for analysis contained 2 days of cell secreted factors (i.e. over days 5–7). As cell‐secreted factors such as TGF‐β2 can become bound to the extracellular matrix (i.e. latent TGF‐β complexes) upon secretion, the fibrin hydrogel was digested utilizing nattokinase as described above. To release TGF‐β2 from the latent complex, conditioned media was adjusted to pH 5 with hydrochloric acid. Microfluidic‐based ELISA was performed as previously described in detail in Tan et al. [104] Inflammatory cytokine detection array membrane was performed per manufacturer protocol (R&D Systems, Proteome Profiler Human Cytokine Array, #ARY005B). All samples were stored at −80°C and thawed immediately prior to microfluidic ELISA or cytokine assay.

### 4.15 Lentiviral‐Mediated Gene Expression

The TGFβR2‐mEmerald fusion protein was assembled into a lentiviral pRRL vector at the KpnI and EcoRI sites and expressed using a CMV promoter. The TGFβR2 coding sequence was amplified from a HUVEC cDNA library using forward primer 5’‐TCTATGACGAGCAGCGG‐3’ and reverse primer 5’‐ CTATTTGGTAGTGTTTAGGGAG‐3. The mEmerald coding sequence was a gift from M. Davidson's lab. DNA fragments were assembled using NEBuilder HiFi DNA Assembly Master Mix. Lentivirus was generated in HEK‐293T cells as previously described [105].

### 4.16 VE‐Cadherin BioID

VE‐cadherin‐BioID constructs were generated previously and transduced into hMVECs (Lonza) as previously described [106]. hMVECs stably expressing VE‐cadherin‐BirA* or cytoplasmic (non‐fusion‐tagged) BirA* were generated via lentiviral transduction. For each condition, EGM2‐MV containing 50 µm biotin was added to two confluent 15 cm plates for 20 h. After incubation, hMVEC monolayers were lysed in RIPA buffer (pH 7.4, 50 mm Tris, 150 mm NaCl, 1% v/v Triton‐X, 0.1% w/v sodium dodecyl sulfate, 0.5% w/v sodium deoxycholate) and biotinylated proteins were extracted by incubating lysates with Dynabeads MyOne Streptavidin C1 (Invitrogen) beads for 1 h at room temperature. Beads were washed three times in RIPA buffer and biotinylated proteins were denatured and eluted in 2X sample buffer containing biotin at 95°C for 10 min. Samples were isolated by gel top SDS‐PAGE extraction. In‐gel tryptic digestion was performed, and peptides were desalted using POROS Oligo R3 beads (Thermo Fisher Scientific). Peptides were washed with 0.1% formic acid and peptides were eluted with 50% acetonitrile and 0.1% formic acid. Peptides were dried via lyophilization and resuspended in 5% acetonitrile and 0.1% formic acid before analysis by liquid chromatography‐tandem mass spectrometry (LC‐MS/MS) by MS Bioworks.

### 4.17 Fluorescent Staining

Samples were fixed with 4% paraformaldehyde and permeabilized with a PBS solution containing Triton X‐100 (5% v/v), sucrose (10% w/v), and magnesium chloride (0.6% w/v) for 1 h each at room temperature. AlexaFluor 488 phalloidin (Life Technologies, Carlsbad, CA) was utilized to visualize F‐actin. 4’, 6‐diamidino‐2‐phenylindole (DAPI, 1 µg ml^−1^) was utilized to visualize cell nucleus. To visualize VE‐cadherin, YAP, SNAI1, vimentin, SMAD3, or cleaved caspase3, samples were sequentially blocked in bovine serum albumin (0.3% w/v), incubated with primary antibody [mouse monoclonal anti‐VE‐cadherin (1:500, Santa Cruz Biotechnology, sc‐9989), mouse monoclonal anti‐YAP (1:500, Santa Cruz Biotechnology, sc101199), rabbit monoclonal anti‐SNAI1 (1:500, Cell Signaling Technologies, #3879S), mouse monoclonal anti‐vimentin (1:500, Sigma, V63890), rabbit monoclonal anti‐SMAD3 (1:500, Cell Signaling Technologies, 9523S), or rabbit polyclonal anti‐Caspase‐3 antibody (1:100, Invitrogen)], and incubated with secondary AlexaFluor 647 goat anti‐mouse or anti‐rabbit IgG (H+L) (1:1000, Life Technologies) each for 1 h at room temperature. For TGF‐βR inhibition studies, apoptosis was visualized by CellEvent Caspase‐3/7 Detection Reagent (5 µm, Invitrogen, C10423) added to samples one hour prior to fixation. For live cell VE‐cadherin pulse studies, a mouse monoclonal anti‐VE‐cadherin antibody (55‐7H1 clone) pre‐conjugated with AlexaFluor 647 was added to cell culture media (1:500, BD Biosciences, 561567) for 30 min followed by media rinses as described in [107].

For TGFβR2‐AJ localization studies, MVECs were transduced with TGFβR2‐mEmerald lentivirus and were seeded on glass coverslips coated with 10 µg/ml fibronectin (Fisher CB40008A) or on fibers functionalized with RGD as described above. Cells were fixed with 4% PFA in PBS for 10 min at 37°C. Samples were permeabilized with 0.1% Triton X‐100 at room temperature for 10 min and blocked in 2% w/v BSA in PBS at room temperature for at least one hour. Samples were incubated with mouse anti‐VE‐cadherin (1:300; F‐8; Santa Cruz Biotechnology) primary antibody diluted in blocking buffer, washed with PBS, and subsequently incubated with AlexaFluor647 goat anti‐mouse IgG (1:300) diluted in blocking buffer for one hour at room temperature. Samples were mounted using ProLong Gold Antifade Mountant prior to imaging.

### 4.18 Microscopy and Image Analysis

Fluorescent images were captured on a Zeiss LSM800 confocal microscope. To quantify tip cell formation, the number and distance of tip cells were measured in FIJI. Tip cells were defined morphologically as the leading cell of an invading strand or as a single invading cell. VE‐cadherin signal intensity was quantified by summing the total VE‐cadherin signal and normalizing to the number of cells in each field of view. Nuclear SMAD3 intensity was quantified by first masking the SMAD3 signal with a nuclear mask, then summing SMAD3 intensity and normalizing to the number of cells in each field of view. Performing these analyses on a field of view basis allowed for Pearson's correlation analysis between nuclear SMAD3 and VE‐cadherin. Measurements of VE‐cadherin junctional width was performed as described previously [62, 64, 108]. Briefly, high resolution images of VE‐cadherin were acquired on a confocal microscope. Images across conditions were thresholded under the same parameters. A line orthogonal to the long axis of the junction was drawn to measure the intensity profile and obtain VE‐cadherin junction width.

TGFβR2‐mEmerald immunofluorescent staining was imaged using a Yokogawa CSU10 spinning disk confocal on a Nikon TE2000 microscope with a Cool‐SNAP HQ2 camera (Photometrics), controlled by NIS Elements software (Nikon). To determine TGFβR2 junctional enrichment, an image threshold was generated using the VE‐cadherin signal. TGFβR2‐mEmerald intensity was calculated within and outside the VE‐cadherin threshold area and the ratio of junctional to cytoplasmic intensity was calculated.

### 4.19 Statistics

Statistical significance was determined by one‐way analysis of variance (ANOVA) or two‐sided student's t‐test where appropriate, with significance indicated by *p*<0.05. Sample size is indicated within corresponding figure legends and all data are presented as mean ± standard deviation.

## Author Contributions

W.Y.W., J.X., K.A.J., M.L.K., and B.M.B. designed all of the experiments. W.Y.W., K.A.J., E.H.J., and D.L. conducted in vitro experiments and data analysis. J.X., R.N.K. III, E.H.S., A.S., and B.M.B. carried out in vivo studies and data analysis. K.L. and C.P. provided support for imaging of western blots. H.L.H. and C.D.D. performed mechanical testing on hydrogels. X.T. and X.F. performed microfluidic ELISA measurements. K.A.J. and M.L.K. performed VE‐cadherin BioID, VE‐cadherin CRISPR, TGF‐βR2 experiments. W.Y.W., J.X., M.L.K., and B.M.B. wrote the manuscript. All authors reviewed and edited the manuscript.

## Conflicts of Interest

The authors declare no conflicts of interest.

## Supporting information




**Supporting File 1**: advs75976‐sup‐0001‐SuppMat.docx.

**Supporting File 2**: advs75976‐sup‐0002‐MovieS1‐S5.zip.

## Data Availability

The data that support the findings of this study are available from the corresponding authors upon request.

## References

[1] M. Zhao , L. Wang , M. Wang , et al., “Targeting Fibrosis: Mechanisms and Clinical Trials,” Signal Transduction and Targeted Therapy 7 (2022): 1–21, 10.1038/s41392-022-01070-3. 35773269 PMC9247101

[2] C. Hanumegowda , L. Farkas , and M. Kolb , “Angiogenesis in Pulmonary Fibrosis,” Chest 142 (2012): 200–207, 10.1378/chest.11-1962. 22796840

[3] S. Barratt and A. Millar , “Vascular Remodelling in the Pathogenesis of Idiopathic Pulmonary Fibrosis,” QJM: An International Journal of Medicine 107 (2014): 515–519, 10.1093/qjmed/hcu012. 24453283 PMC4071293

[4] M. Ebina , M. Shimizukawa , N. Shibata , et al., “Heterogeneous Increase in CD34‐positive Alveolar Capillaries in Idiopathic Pulmonary Fibrosis,” American Journal of Respiratory and Critical Care Medicine 169 (2004): 1203–1208, 10.1164/rccm.200308-1111OC. 14754760

[5] S. A. Jimenez and S. Piera‐Velazquez , “Endothelial to Mesenchymal Transition (EndoMT) in the Pathogenesis of Systemic Sclerosis‐associated Pulmonary Fibrosis and Pulmonary Arterial Hypertension. Myth or Reality?,” Matrix Biology 51 (2016): 26–36, 10.1016/j.matbio.2016.01.012. 26807760 PMC4842122

[6] N. W. Hultgren , J. S. Fang , M. E. Ziegler , et al., “Slug Regulates the Dll4‐Notch‐VEGFR2 Axis to Control Endothelial Cell Activation and Angiogenesis,” Nature Communications 11 (2020): 1–16, 10.1038/s41467-020-18633-z. PMC7588439 33106502

[7] K. M. Welch‐Reardon , N. Wu , and C. C. W. Hughes , “A Role for Partial Endothelial–Mesenchymal Transitions in Angiogenesis?,” Arteriosclerosis, Thrombosis, and Vascular Biology 35 (2015): 303–308, 10.1161/ATVBAHA.114.303220. 25425619 PMC4911209

[8] S. Piera‐Velazquez , Z. Li , and S. A. Jimenez , “Role of Endothelial‐mesenchymal Transition (EndoMT) in the Pathogenesis of Fibrotic Disorders,” The American Journal of Pathology 179 (2011): 1074–1080, 10.1016/j.ajpath.2011.06.001. 21763673 PMC3157273

[9] E. Dejana , K. K. Hirschi , and M. Simons , “The Molecular Basis of Endothelial Cell Plasticity,” Nature Communications 8 (2017): 1–11, 10.1038/ncomms14361. PMC5309780 28181491

[10] E. M. Zeisberg , O. Tarnavski , M. Zeisberg , et al., “Endothelial‐to‐mesenchymal Transition Contributes to Cardiac Fibrosis,” Nature Medicine 13 (2007): 952–961, 10.1038/nm1613. 17660828

[11] S. Potenta , E. Zeisberg , and R. Kalluri , “The Role of Endothelial‐to‐Mesenchymal Transition in Cancer Progression,” British Journal of Cancer 99 (2008): 1375–1379, 10.1038/sj.bjc.6604662. 18797460 PMC2579683

[12] Z. Cao , T. Ye , Y. Sun , et al., “Targeting the Vascular and Perivascular Niches as a Regenerative Therapy for Lung and Liver Fibrosis,” Science Translational Medicine 9 (2017): eaai8710, 10.1126/scitranslmed.aai8710. 28855398 PMC5606244

[13] Z. Cao , R. Lis , M. Ginsberg , et al., “Targeting of the Pulmonary Capillary Vascular Niche Promotes Lung Alveolar Repair and Ameliorates Fibrosis,” Nature Medicine 22 (2016): 154–162, 10.1038/nm.4035. PMC4872630 26779814

[14] B.‐S. Ding , Z. Cao , R. Lis , et al., “Divergent Angiocrine Signals From Vascular Niche Balance Liver Regeneration and Fibrosis,” Nature 505 (2014): 97–102, 10.1038/nature12681. 24256728 PMC4142699

[15] R. Del Toro , et al., “Identification and Functional Analysis of Endothelial Tip Cell‐Enriched Genes,” Blood 116 (2010): 4025–4033, 10.1182/blood-2010-02-270819. 20705756 PMC4314527

[16] K. M. Welch‐Reardon , et al., “Angiogenic Sprouting is Regulated by Endothelial Cell Expression of Slug,” Journal of Cell Science 127 (2014): 2017–2028. 24554431 10.1242/jcs.143420 PMC4004976

[17] J.‐X. Sun , T.‐F. Chang , M.‐H. Li , et al., “SNAI1, an Endothelial–Mesenchymal Transition Transcription Factor, Promotes the Early Phase of Ocular Neovascularization,” Angiogenesis 21 (2018): 635–652, 10.1007/s10456-018-9614-9. 29675549

[18] A. Vannan , R. Lyu , A. L. Williams , et al., “Spatial Transcriptomics Identifies Molecular Niche Dysregulation Associated With Distal Lung Remodeling in Pulmonary Fibrosis,” Nature Genetics 57 (2025): 647–658, 10.1038/s41588-025-02080-x. 39901013 PMC11906353

[19] N. Hashimoto , S. H. Phan , K. Imaizumi , et al., “Endothelial–Mesenchymal Transition in Bleomycin‐Induced Pulmonary Fibrosis,” American Journal of Respiratory Cell and Molecular Biology 43 (2010): 161–172, 10.1165/rcmb.2009-0031OC. 19767450 PMC2937229

[20] R. Kalluri and M. Zeisberg , “Fibroblasts in Cancer,” Nature Reviews Cancer 6 (2006): 392–401, 10.1038/nrc1877. 16572188

[21] L. A. Van Meeteren and P. Ten Dijke , “Regulation of Endothelial Cell Plasticity by TGF‐β,” Cell and Tissue Research 347 (2012): 177–186. 21866313 10.1007/s00441-011-1222-6 PMC3250609

[22] P. Cipriani , P. Di Benedetto , P. Ruscitti , et al., “The Endothelial‐mesenchymal Transition in Systemic Sclerosis is Induced by Endothelin‐1 and Transforming Growth Factor‐β and may be Blocked by Macitentan, a Dual Endothelin‐1 Receptor Antagonist,” The Journal of Rheumatology 42 (2015): 1808–1816, 10.3899/jrheum.150088. 26276964

[23] M. Maleszewska , J.‐R. A. J. Moonen , N. Huijkman , B. van de Sluis , G. Krenning , and M. C. Harmsen , “IL‐1β and TGFβ2 Synergistically Induce Endothelial to Mesenchymal Transition in an NFκB‐dependent Manner,” Immunobiology 218 (2013): 443–454, 10.1016/j.imbio.2012.05.026. 22739237

[24] M. Manetti , E. Romano , I. Rosa , et al., “Endothelial‐to‐mesenchymal Transition Contributes to Endothelial Dysfunction and Dermal Fibrosis in Systemic Sclerosis,” Annals of the Rheumatic Diseases 76 (2017): 924–934, 10.1136/annrheumdis-2016-210229. 28062404

[25] F. A. Mendoza , S. Piera‐Velazquez , J. L. Farber , C. Feghali‐Bostwick , and S. A. Jiménez , “Endothelial Cells Expressing Endothelial and Mesenchymal Cell Gene Products in Lung Tissue From Patients With Systemic Sclerosis–Associated Interstitial Lung Disease,” Arthritis & Rheumatology 68 (2016): 210–217, 10.1002/art.39421. 26360820 PMC4690777

[26] Jefferson Digital Commons , “Endothelial Cells Expressing Endothelial and Mesenchymal Cell Endothelial Cells Expressing Endothelial and Mesenchymal Cell Gene Products in Lung Tissue from Patients with Systemic Gene Products in Lung Tissue from Patients with Systemic Sclerosis‐Associated Interstitial Lung Disease. Sclerosis‐Associated Interstitial Lung Disease. Endothelial Cells Expressing Endothelial and Mesenchymal Cell Gene Products in Lung Tissue from Patients with Systemic,” (2016) https://jdc.jefferson.edu/dcbfp. 10.1002/art.39421 PMC4690777 26360820

[27] S. C. Wei , L. Fattet , J. H. Tsai , et al., “Matrix Stiffness Drives Epithelial–mesenchymal Transition and Tumour Metastasis through a TWIST1–G3BP2 Mechanotransduction Pathway,” Nature Cell Biology 17 (2015): 678–688, 10.1038/ncb3157. 25893917 PMC4452027

[28] J. Yang and R. A. Weinberg , “Epithelial‐Mesenchymal Transition: at the Crossroads of Development and Tumor Metastasis,” Developmental Cell 14 (2008): 818–829, 10.1016/j.devcel.2008.05.009. 18539112

[29] S. C. Wei and J. Yang , “Forcing through Tumor Metastasis: the Interplay between Tissue Rigidity and Epithelial–Mesenchymal Transition,” Trends in Cell Biology 26 (2016): 111–120, 10.1016/j.tcb.2015.09.009. 26508691 PMC4728004

[30] S. C. Wei , L. Fattet , and J. Yang , “The Forces behind EMT and Tumor Metastasis,” Cell Cycle 14 (2015): 2387–2388, 10.1080/15384101.2015.1063296. 26083471 PMC5038851

[31] C. J. Shen , S. Raghavan , Z. Xu , et al., “Decreased Cell Adhesion Promotes Angiogenesis in a Pyk2‐Dependent Manner,” Experimental Cell Research 317 (2011): 1860–1871, 10.1016/j.yexcr.2011.05.006. 21640103 PMC3123418

[32] J. S. Miller , C. J. Shen , W. R. Legant , J. D. Baranski , B. L. Blakely , and C. S. Chen , “Bioactive Hydrogels Made From Step‐Growth Derived PEG–Peptide Macromers,” Biomaterials 31 (2010): 3736–3743, 10.1016/j.biomaterials.2010.01.058. 20138664 PMC2837100

[33] B. Trappmann , B. M. Baker , W. J. Polacheck , C. K. Choi , J. A. Burdick , and C. S. Chen , “Matrix Degradability Controls Multicellularity of 3D Cell Migration,” Nature Communications 8 (2017): 1–8, 10.1038/s41467-017-00418-6. PMC5575316 28851858

[34] B. N. Mason , A. Starchenko , R. M. Williams , L. J. Bonassar , and C. A. Reinhart‐King , “Tuning Three‐Dimensional Collagen Matrix Stiffness Independently of Collagen Concentration Modulates Endothelial Cell Behavior,” Acta Biomaterialia 9 (2013): 4635–4644, 10.1016/j.actbio.2012.08.007. 22902816 PMC3508162

[35] L. Schimmel and E. Gordon , “The Precise Molecular Signals That Control Endothelial Cell–Cell Adhesion Within the Vessel Wall,” Biochemical Society Transactions 46 (2018): 1673–1680, 10.1042/BST20180377. 30514769 PMC6299237

[36] Y. Blum , H.‐G. Belting , E. Ellertsdottir , L. Herwig , F. Lüders , and M. Affolter , “Complex Cell Rearrangements During Intersegmental Vessel Sprouting and Vessel Fusion in the Zebrafish Embryo,” Developmental Biology 316 (2008): 312–322, 10.1016/j.ydbio.2008.01.038. 18342303

[37] K. Matsumoto , T. T. Mitani , S. A. Horiguchi , et al., “Advanced CUBIC Tissue Clearing for Whole‐organ Cell Profiling,” Nature Protocols 14 (2019): 3506–3537, 10.1038/s41596-019-0240-9. 31748753

[38] A. M. Taylor and B. Bordoni , Histology, Blood Vascular System (StatPearls, 2023), https://www.ncbi.nlm.nih.gov/books/NBK553217/. 31985998

[39] A. S. Patel , A. Smith , S. Nucera , et al., “TIE2‐expressing Monocytes/Macrophages Regulate Revascularization of the Ischemic Limb,” EMBO Molecular Medicine 5 (2013): 858–869, 10.1002/emmm.201302752. 23653322 PMC3779448

[40] S. Payne , S. D. Val , and A. Neal , “Endothelial‐Specific Cre Mouse Models: is your cre Credibile?,” Arteriosclerosis, Thrombosis, and Vascular Biology 38 (2018): 2550–2561, 10.1161/ATVBAHA.118.309669. 30354251 PMC6218004

[41] W. Y. Wang , E. H. Jarman , D. Lin , and B. M. Baker , “Dynamic Endothelial Stalk Cell–Matrix Interactions Regulate Angiogenic Sprout Diameter,” Frontiers in Bioengineering and Biotechnology 9 (2021): 187. 10.3389/fbioe.2021.620128 PMC8044977 33869150

[42] W. Y. Wang , R. N. Kent , S. A. Huang , et al., “Direct Comparison of Angiogenesis in Natural and Synthetic Biomaterials Reveals That Matrix Porosity Regulates Endothelial Cell Invasion Speed and Sprout Diameter,” Acta Biomaterialia 135 (2021): 260–273, 10.1016/j.actbio.2021.08.038. 34469789 PMC8595798

[43] W. Y. Wang , D. Lin , E. H. Jarman , W. J. Polacheck , and B. M. Baker , “Functional Angiogenesis Requires Microenvironmental Cues Balancing Endothelial Cell Migration and Proliferation,” Lab on a Chip 20 (2020): 1153–1166, 10.1039/C9LC01170F. 32100769 PMC7328820

[44] D. L. Matera , A. T. Lee , H. L. Hiraki , and B. M. Baker , “The Role of Rho GTPases during Fibroblast Spreading, Migration, and Myofibroblast Differentiation in 3D Synthetic Fibrous Matrices,” Cellular and Molecular Bioengineering 14 (2021): 381–396, 10.1007/s12195-021-00698-5. 34777599 PMC8548490

[45] D. L. Matera , K. M. DiLillo , M. R. Smith , et al., “Microengineered 3D Pulmonary Interstitial Mimetics Highlight a Critical Role for Matrix Degradation in Myofibroblast Differentiation,” Science Advances 6 (2020): eabb5069, 10.1126/sciadv.abb5069. 32917680 PMC11206459

[46] D. L. Matera , W. Y. Wang , M. R. Smith , A. Shikanov , and B. M. Baker , “Fiber Density Modulates Cell Spreading in 3D Interstitial Matrix Mimetics,” ACS Biomaterials Science & Engineering 5 (2019): 2965–2975, 10.1021/acsbiomaterials.9b00141. 33405599

[47] M. G. Tonnesen , X. Feng , and R. A. F. F. Clark , “Angiogenesis in Wound Healing,” Journal of Investigative Dermatology Symposium Proceedings 5 (2000): 40–46, 10.1046/j.1087-0024.2000.00014.x. 11147674

[48] C. K. Probst , S. B. Montesi , B. D. Medoff , B. S. Shea , and R. S. Knipe , “Vascular Permeability in the Fibrotic Lung,” European Respiratory Journal 56 (2020): 1–17, 10.1183/13993003.00100-2019. PMC9977144 32265308

[49] J. H. Paik , C. Ss , M. J. Lee , S. Thangada , and T. Hla , “Sphingosine 1‐Phosphate‐Induced Endothelial Cell Migration Requires the Expression of EDG‐1 and EDG‐3 Receptors and Rho‐Dependent Activation of αvβ3‐ and β1‐Containing Integrins,” Journal of Biological Chemistry 276 (2001): 11830–11837, 10.1074/jbc.M009422200. 11150298

[50] H. A. Belcher , M. Guthold , and N. E. Hudson , “What is the Diameter of a Fibrin fiber?,” Research and Practice in Thrombosis and Haemostasis 7 (2023): 100285, 10.1016/j.rpth.2023.100285. 37601015 PMC10439396

[51] C. D. Davidson , D. K. P. Jayco , D. L. Matera , et al., “Myofibroblast Activation in Synthetic Fibrous Matrices Composed of Dextran Vinyl Sulfone,” Acta Biomaterialia 105 (2020): 78–86, 10.1016/j.actbio.2020.01.009. 31945504 PMC7369643

[52] K. M. Riching , B. L. Cox , M. R. Salick , et al., “3D Collagen Alignment Limits Protrusions to Enhance Breast Cancer Cell Persistence,” Biophysical Journal 107 (2015): 2546–2558, 10.1016/j.bpj.2014.10.035. PMC4255204 25468334

[53] X. Gong , J. Kulwatno , and K. L. Mills , “Rapid Fabrication of Collagen Bundles Mimicking Tumor‐Associated Collagen Architectures,” Acta Biomaterialia 108 (2020): 128–141, 10.1016/j.actbio.2020.03.019. 32194262

[54] L. E. Scott , S. H. Weinberg , and C. A. Lemmon , “Mechanochemical Signaling of the Extracellular Matrix in Epithelial‐Mesenchymal Transition,” Frontiers in Cell and Developmental Biology 7 (2019): 449156, 10.3389/fcell.2019.00135. PMC6658819 31380370

[55] H. L. Hiraki , D. L. Matera , W. Y. Wang , et al., “Fiber Density and Matrix Stiffness Modulate Distinct Cell Migration Modes in a 3D Stroma Mimetic Composite Hydrogel,” Acta Biomaterialia 163 (2023): 378–391, 10.1016/j.actbio.2022.09.043. 36179980 PMC10043045

[56] L. A. Van Meeteren and P. Ten Dijke , “Regulation of Endothelial Cell Plasticity by TGF‐β,” Cell and Tissue Research 347 (2011): 177. 21866313 10.1007/s00441-011-1222-6 PMC3250609

[57] S. Mascharak , P. L. Benitez , A. C. Proctor , et al., “YAP‐dependent Mechanotransduction is Required for Proliferation and Migration on Native‐Like Substrate Topography,” Biomaterials 115 (2016): 155–166, 10.1016/j.biomaterials.2016.11.019. 27889666 PMC5572766

[58] T. Toyama , A. P. Looney , B. M. Baker , et al., “Therapeutic Targeting of TAZ and YAP by Dimethyl Fumarate in Systemic Sclerosis Fibrosis,” Journal of Investigative Dermatology 138 (2018): 78–88, 10.1016/j.jid.2017.08.024. 28870693 PMC5742036

[59] F. Orsenigo , C. Giampietro , A. Ferrari , et al., “Phosphorylation of VE‐cadherin is Modulated by Haemodynamic Forces and Contributes to the Regulation of Vascular Permeability in Vivo,” Nature Communications 3 (2012): 1–15, 10.1038/ncomms2199. PMC3514492 23169049

[60] S. J. Depalma , C. D. Davidson , A. E. Stis , A. S. Helms , and B. M. Baker , “Microenvironmental Determinants of Organized iPSC‐cardiomyocyte Tissues on Synthetic Fibrous Matrices,” Biomaterials Science 9 (2021): 93–107, 10.1039/D0BM01247E. 33325920 PMC7971708

[61] Y. L. Dorland , T. S. Malinova , A.‐M. D. van Stalborch , et al., “The F‐BAR Protein pacsin2 Inhibits Asymmetric VE‐cadherin Internalization From Tensile Adherens Junctions,” Nature Communications 7 (2016): 1–18, 10.1038/ncomms12210. PMC4947187 27417273

[62] J. Huynh , N. Nishimura , K. Rana , et al., “Age‐Related Intimal Stiffening Enhances Endothelial Permeability and Leukocyte Transmigration,” Science Translational Medicine 3 (2011): 112ra122–112ra122, 10.1126/scitranslmed.3002761. PMC3693751 22158860

[63] D. Mehta and A. B. Malik , “Signaling Mechanisms Regulating Endothelial Permeability,” Physiological Reviews 86 (2006): 279–367, 10.1152/physrev.00012.2005. 16371600

[64] G. P. Van Nieuw Amerongen , C. M. L. Beckers , I. D. Achekar , S. Zeeman , R. J. P. Musters , and V. W. M. van Hinsbergh , “Involvement of Rho Kinase in Endothelial Barrier Maintenance,” Arteriosclerosis, Thrombosis, and Vascular Biology 27 (2007): 2332–2339. 17761936 10.1161/ATVBAHA.107.152322

[65] C. D. Davidson , F. S. Midekssa , S. J. DePalma , et al., “Mechanical Intercellular Communication via Matrix‐Borne Cell Force Transmission during Vascular Network Formation,” Advanced Science 11 (2024): 2306210, 10.1002/advs.202306210. 37997199 PMC10797481

[66] F. S. Midekssa , C. D. Davidson , M. E. Wieger , et al., “Semi‐Synthetic Fibrous Fibrin Composites Promote 3D Microvascular Assembly, Survival, and Host Integration of Endothelial Cells Without Mesenchymal Cell Support,” Bioactive Materials 49 (2025): 652–669. 40235652 10.1016/j.bioactmat.2025.02.029 PMC11999628

[67] D. Medici , S. Potenta , and R. Kalluri , “Transforming Growth Factor‐β2 Promotes Snail‐Mediated Endothelial–mesenchymal Transition Through Convergence of Smad‐Dependent and Smad‐Independent Signalling,” Biochemical Journal 437 (2011): 515–520, 10.1042/BJ20101500. 21585337 PMC4457510

[68] E. Dejana , “Endothelial Cell–Cell Junctions: Happy Together,” Nature Reviews Molecular Cell Biology 5 (2004): 261–270, 10.1038/nrm1357. 15071551

[69] E. Siljamäki , P. Riihilä , U. Suwal , et al., “Inhibition of TGF‐β Signaling, Invasion, and Growth of Cutaneous Squamous Cell Carcinoma by PLX8394,” Oncogene 42 (2023): 3633–3647, 10.1038/s41388-023-02863-8. 37864034 PMC10691969

[70] A. Weiss and L. Attisano , “The TGFbeta Superfamily Signaling Pathway,” WIREs Developmental Biology 2 (2013): 47–63, 10.1002/wdev.86. 23799630

[71] J. L. Leight , M. A. Wozniak , S. Chen , M. L. Lynch , and C. S. Chen , “Matrix Rigidity Regulates a Switch Between TGF‐β1–Induced Apoptosis and Epithelial–mesenchymal Transition,” Molecular Biology of the Cell 23 (2012): 781–791, 10.1091/mbc.e11-06-0537. 22238361 PMC3290638

[72] S. F. Mohammed , S. Hussain , S. A. Mirzoyev , W. D. Edwards , J. J. Maleszewski , and M. M. Redfield , “Coronary Microvascular Rarefaction and Myocardial Fibrosis in Heart Failure with Preserved Ejection Fraction,” Circulation 131 (2015): 550–559, 10.1161/CIRCULATIONAHA.114.009625. 25552356 PMC4324362

[73] M. S. Goligorsky , “Microvascular Rarefaction: The Decline and Fall of Blood Vessels,” Organogenesis 6 (2010): 1–10. 20592859 10.4161/org.6.1.10427 PMC2861737

[74] C. M. Magro , W. J. Waldman , D. A. Knight , et al., “Idiopathic Pulmonary Fibrosis Related to Endothelial Injury and Antiendothelial Cell Antibodies,” Human Immunology 67 (2006): 284–297, 10.1016/j.humimm.2006.02.026. 16720208

[75] M. Potente , H. Gerhardt , and P. Carmeliet , “Basic and Therapeutic Aspects of Angiogenesis,” Cell 146 (2011): 873–887, 10.1016/j.cell.2011.08.039. 21925313

[76] W. Zhang , C.‐H. Lu , M. L. Nakamoto , et al., “Curved Adhesions Mediate Cell Attachment to Soft Matrix Fibres in Three Dimensions,” Nature Cell Biology 25 (2023): 1453–1464, 10.1038/s41556-023-01238-1. 37770566 PMC10567576

[77] E. E. Bastounis , Y. T. Yeh , and J. A. Theriot , “Subendothelial Stiffness Alters Endothelial Cell Traction Force Generation While Exerting a Minimal Effect on the Transcriptome,” Scientific Reports 9 (2019): 1–16, 10.1038/s41598-019-54336-2. 31796790 PMC6890669

[78] A. A. Raslan , T. X. Pham , J. Lee , et al., “Lung Injury‐induced Activated Endothelial Cell States Persist in Aging‐associated Progressive Fibrosis,” Nature Communications 15 (2024): 5449, 10.1038/s41467-024-49545-x. PMC11211333 38937456

[79] E. Engelbrecht , T. Kooistra , N. Burg , et al., “Ectopic Expansion of Pulmonary Vasculature in Fibrotic Lung Disease and Lung Adenocarcinoma Marked by Proangiogenic COL15A1+ Endothelial Cells,” Pulmonary Circulation 15 (2025): 70102, 10.1002/pul2.70102. PMC12130637 40463493

[80] J. C. Schupp , T. S. Adams , C. Cosme , et al., “Integrated Single‐Cell Atlas of Endothelial Cells of the Human Lung,” Circulation 144 (2021): 286–302, 10.1161/CIRCULATIONAHA.120.052318. 34030460 PMC8300155

[81] P. S. Hasleton and D. B. Flieder , Spencer's Pathology of the Lung Vol. 2 (Spencer, 2013), 10.1017/CBO9781139018760.

[82] A. B. Lumb , “Nunn's Applied Respiratory Physiology EBook:& Nunn's Applied Respiratory Physiology EBook,” in Nunn's Applied Respiratory Physiology EBook (Elsevier, 2016).

[83] J. B. Tefft , J. L. Bays , A. Lammers , S. Kim , J. Eyckmans , and C. S. Chen , “Notch1 and Notch3 Coordinate for Pericyte‐induced Stabilization of Vasculature,” American Journal of Physiology‐Cell Physiology 322 (2021): C185–C196, 10.1152/ajpcell.00320.2021. 34878922 PMC8791789

[84] C. M. Franca , M. E. Lima Verde , A. C. Silva‐Sousa , et al., “Perivascular Cells Function as Key Mediators of Mechanical and Structural Changes in Vascular Capillaries,” Science Advances 11 (2025): adp3789, 10.1126/sciadv.adp3789. PMC11721577 39792671

[85] J. R. Johnson , E. Folestad , J. E. Rowley , et al., “Pericytes Contribute to Airway Remodeling in a Mouse Model of Chronic Allergic Asthma,” American Journal of Physiology‐Lung Cellular and Molecular 308 (2015): L658–L671. 10.1152/ajplung.00286.2014 PMC4385988 25637607

[86] R. Bignold , B. Shammout , J. E. Rowley , M. Repici , J. Simms , and J. R. Johnson , “Chemokine CXCL12 Drives Pericyte Accumulation and Airway Remodeling in Allergic Airway Disease,” Respiratory Research 23 (2022): 183. 35831901 10.1186/s12931-022-02108-4 PMC9277926

[87] A. T. Garrison , R. E. Bignold , X. Wu , and J. R. Johnson , “Pericytes: The Lung‐forgotten Cell Type,” Frontiers in Physiology 14 (2023): 1150028, 10.3389/fphys.2023.1150028. 37035669 PMC10076600

[88] M. Crisan , S. Yap , L. Casteilla , et al., “A Perivascular Origin for Mesenchymal Stem Cells in Multiple Human Organs,” Cell Stem Cell 3 (2008): 301–313, 10.1016/j.stem.2008.07.003. 18786417

[89] J. Feng , A. Mantesso , C. De Bari , A. Nishiyama , and P. T. Sharp , “Dual Origin of Mesenchymal Stem Cells Contributing to Organ Growth and Repair,” Proceedings of the National Academy of Sciences 108 (2011): 6503–6508, 10.1073/pnas.1015449108. PMC3081015 21464310

[90] R. R. Rao , A. W. Peterson , J. Ceccarelli , A. J. Putnam , and J. P. Stegemann , “Matrix Composition Regulates Three‐Dimensional Network Formation by Endothelial Cells and Mesenchymal Stem Cells in Collagen/Fibrin Materials,” Angiogenesis 15 (2012): 253–264, 10.1007/s10456-012-9257-1. 22382584 PMC3756314

[91] S. Alimperti , T. Mirabella , V. Bajaj , et al., “Three‐Dimensional Biomimetic Vascular Model Reveals a RhoA, Rac1, and N‐cadherin Balance in Mural Cell–endothelial Cell‐Regulated Barrier Function,” Proceedings of the National Academy of Sciences 114 (2017): 8758–8763, 10.1073/pnas.1618333114. PMC5565405 28765370

[92] R. Kramann , C. Goettsch , J. Wongboonsin , et al., “Adventitial MSC‐Like Cells Are Progenitors of Vascular Smooth Muscle Cells and Drive Vascular Calcification in Chronic Kidney Disease,” Cell Stem Cell 19 (2016): 628–642, 10.1016/j.stem.2016.08.001. 27618218 PMC5097006

[93] R. Haidari , W. J. Fowler , S. D. Robinson , R. T. Johnson , and D. T. Warren , “Microvascular Endothelial Cells Display Organ‐Specific Responses to Extracellular Matrix Stiffness,” Current Research in Physiology 8 (2025): 100140, 10.1016/j.crphys.2025.100140. 39967829 PMC11833412

[94] X. Wu , D. Zhang , X. Qiao , et al., “Regulating the Cell Shift of Endothelial Cell‐Like Myofibroblasts in Pulmonary Fibrosis,” European Respiratory Journal 61 (2023): 2201799, 10.1183/13993003.01799-2022. 36758986 PMC10249020

[95] A. A. Raslan , T. X. Pham , J. Lee , et al., “Lung Injury‐Induced Activated Endothelial Cell States Persist in Aging‐Associated Progressive Fibrosis,” Nature Communications 15 (2024): 1–20, 10.1038/s41467-024-49545-x. PMC11211333 38937456

[96] T. Sun , Z. Huang , W.‐C. Liang , et al., “TGFβ2 and TGFβ3 Isoforms Drive Fibrotic Disease Pathogenesis,” Science Translational Medicine 13 (2021): abe0407, 10.1126/scitranslmed.abe0407. 34349032

[97] M.‐J. Goumans , G. Valdimarsdottir , S. Itoh , A. Rosendahl , P. Sideras , and P. ten Dijke , “Balancing the Activation state of the Endothelium via Two Distinct TGF‐β Type I Receptors,” The EMBO Journal 21 (2002): 1743–1753, 10.1093/emboj/21.7.1743. 11927558 PMC125949

[98] P.‐Y. Chen , L. Qin , G. Li , et al., “Endothelial TGF‐β Signalling Drives Vascular Inflammation and Atherosclerosis,” Nature Metabolism 1 (2019): 912–926, 10.1038/s42255-019-0102-3. PMC6767930 31572976

[99] Y. Yu and Y. Chau , “One‐Step “Click” Method for Generating Vinyl Sulfone Groups on Hydroxyl‐Containing Water‐Soluble Polymers,” Biomacromolecules 13 (2012): 937–942, 10.1021/bm2014476. 22229738

[100] W. J. Polacheck , M. L. Kutys , J. Yang , et al., “A Non‐Canonical Notch Complex Regulates Adherens Junctions and Vascular Barrier Function,” Nature 552 (2017): 258–262, 10.1038/nature24998. 29160307 PMC5730479

[101] W. J. Polacheck , M. L. Kutys , J. B. Tefft , and C. S. Chen , “Microfabricated Blood Vessels for Modeling the Vascular Transport Barrier,” Nature Protocols 14 (2019): 1425–1454, 10.1038/s41596-019-0144-8. 30953042 PMC7046311

[102] R. H. Adamson , J. F. Lenz , and F. E. Curry , “Quantitative Laser Scanning Confocal Microscopy on Single Capillaries: Permeability Measurement,” Microcirculation 1 (1994): 251–265, 10.3109/10739689409146752. 8790594

[103] S. Ahsan and S. Drăghici , “Identifying Significantly Impacted Pathways and Putative Mechanisms With iPathwayGuide,” CP in Bioinformatics 2017 (2017): 1–7. 10.1002/cpbi.24 28654712

[104] X. Tan , M. K. Khaing Oo , Y. Gong , Y. Li , H. Zhu , and X. Fan , “Glass Capillary Based Microfluidic ELISA for Rapid Diagnostics,” The Analyst 142 (2017): 2378–2385, 10.1039/C7AN00523G. 28548141

[105] M. J. White , K. A. Jacobs , T. Singh , et al., “Notch1 Cortical Signaling Regulates Epithelial Architecture and Cell–Cell Adhesion,” Journal of Cell Biology 222 (2023): 202303013, 10.1083/jcb.202303013. PMC10555887 37796194

[106] L. N. Mayo , F. Duong , A. Mompeón , K. A. Jacobs , M. L. Iruela‐Arispe , and M. L. Kutys , “Scrib Organizes Cortical Actomyosin Clusters to Maintain Adherens Junctions and Angiogenic Sprouting,” BioRxiv (2025): 645927, 10.1101/2025.04.01.645927. 42043432

[107] F. Neto , A. Klaus‐Bergmann , Y. T. Ong , et al., “YAP and TAZ Regulate Adherens Junction Dynamics and Endothelial Cell Distribution During Vascular Development,” Elife 7 (2018): 1–30, 10.7554/eLife.31037. PMC5814147 29400648

[108] F. Bordeleau , B. N. Mason , E. M. Lollis , et al., “Matrix Stiffening Promotes a Tumor Vasculature Phenotype,” Proceedings of the National Academy of Sciences 114 (2017): 492–497, 10.1073/pnas.1613855114. PMC5255592 28034921

