# Supplementary material for: Perivascular Matrix Densification Dysregulates Angiogenesis and Activates Pro‐Inflammatory Endothelial Cells
Source: Adv Sci (Weinh). 2026 Jun 17:e10448. Online ahead of print. doi: 10.1002/advs.202510448 (PMC13336811; doi:10.1002/advs.202510448)
Supplement: Supplementary file 1 — Supporting File 1: advs75976‐sup‐0001‐SuppMat.docx. [file ADVS-9999-e10448-s002.docx]

**Perivascular matrix densification dysregulates angiogenesis and activates pro-inflammatory endothelial cells**

Jingyi Xia^1^*, William Y. Wang^1^*, Kyle A. Jacobs^8^, Kairav Maniar^1^, Daphne Lin^2^, Evan H. Jarman^1^, Daniel L. Matera^2^, Kristen Loesel^3^, Christopher D. Davidson^1^, Harrison L. Hiraki^1^, Xiaotian Tan^1^, Eve H. Shikanov^1^, Robert N. Kent^1^, Carole Parent^3,4,5^, Xudong Fan^1^, Ariella Shikanov^1,6,7^, Matthew L. Kutys^8,+^, Brendon M. Baker^1,+^

^1^Department of Biomedical Engineering

University of Michigan, Ann Arbor, MI 48109

^2^Department of Chemical Engineering

University of Michigan, Ann Arbor, MI 48109

^3^Department of Cell and Developmental Biology

University of Michigan, Ann Arbor, MI 48109

^4^Department of Pharmacology

University of Michigan, Ann Arbor, MI 48109

^5^Rogel Cancer Center Michigan Medicine

University of Michigan, Ann Arbor, MI 48109

^6^Department of Macromolecular Science & Engineering

University of Michigan, Ann Arbor, MI 48109

^7^Department of Obstetrics and Gynecology

University of Michigan, Ann Arbor, MI 48109

^8^Department of Cell and Tissue Biology

University of California San Francisco, San Francisco, CA 94143

* authors contributed equally to this work

^+^ Co-corresponding Authors:

Brendon M. Baker, Ph.D.

Department of Biomedical Engineering, University of Michigan

2174 Lurie BME Building, 1101 Beal Avenue

Ann Arbor, MI 48109

Email: [bambren@umich.edu](mailto:bambren@umich.edu)

Matthew L. Kutys, Ph. D.

Department of Cell and Tissue Biology

513 Parnassus Ave.

University of California San Francisco, San Francisco, CA 94143

Email: Matthew.Kutys@UCSF.edu

**SUPPLEMENTARY MATERIAL**

The Supplementary Material includes 12 Supplementary Figures.


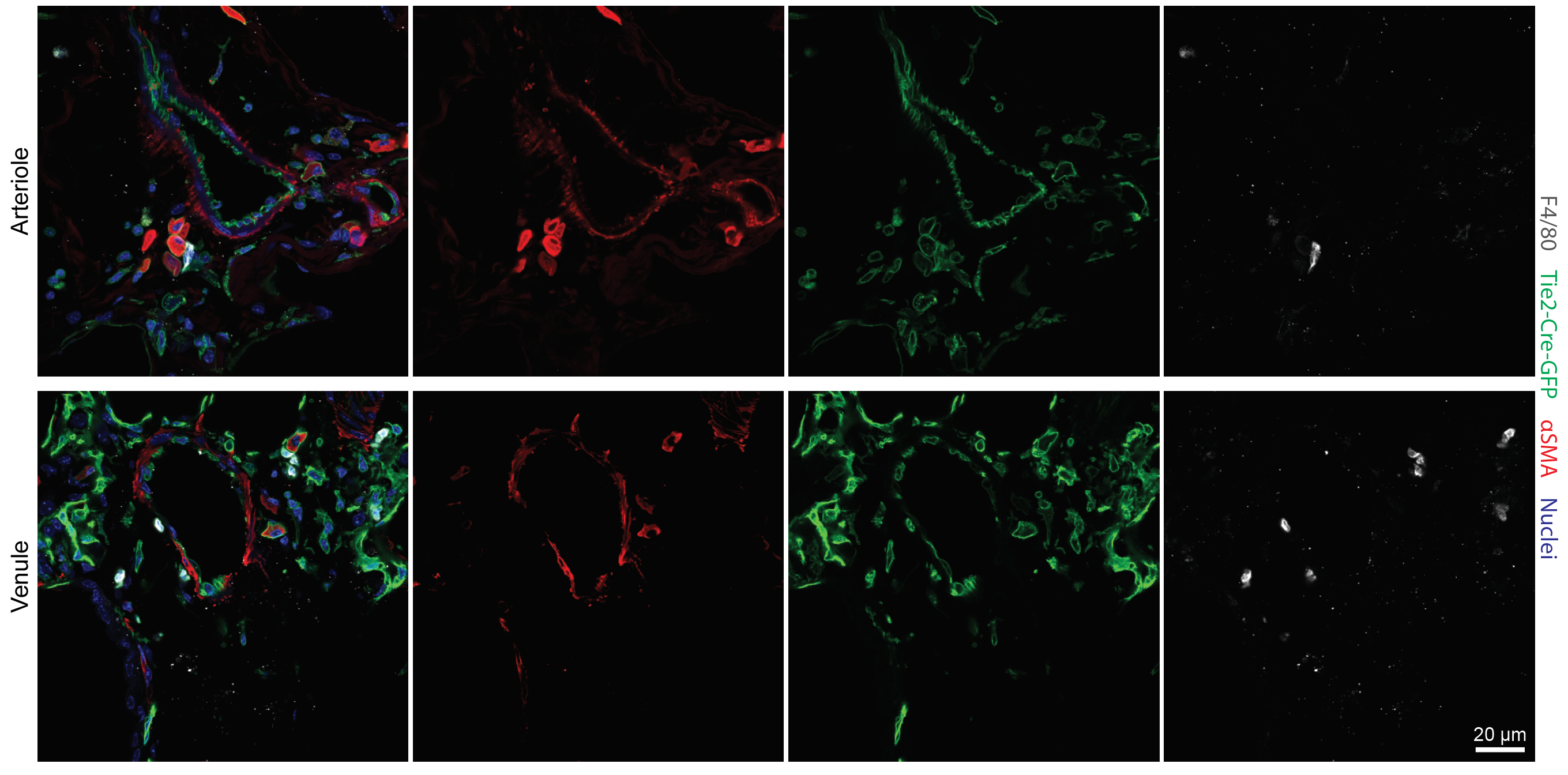


**Supplementary Figure 1:** **Tie2-GFP^+^/αSMA^+^ ECs are a distinct population from F4/80^+^ macrophages.** Representative images of an arteriole (top row, single z-slice from Supplementary Movie 4) and venule (bottom row, single z-slice from Supplementary Movie 5) and surrounding perivascular space from murine lungs 3 weeks after bleomycin-induced injury.


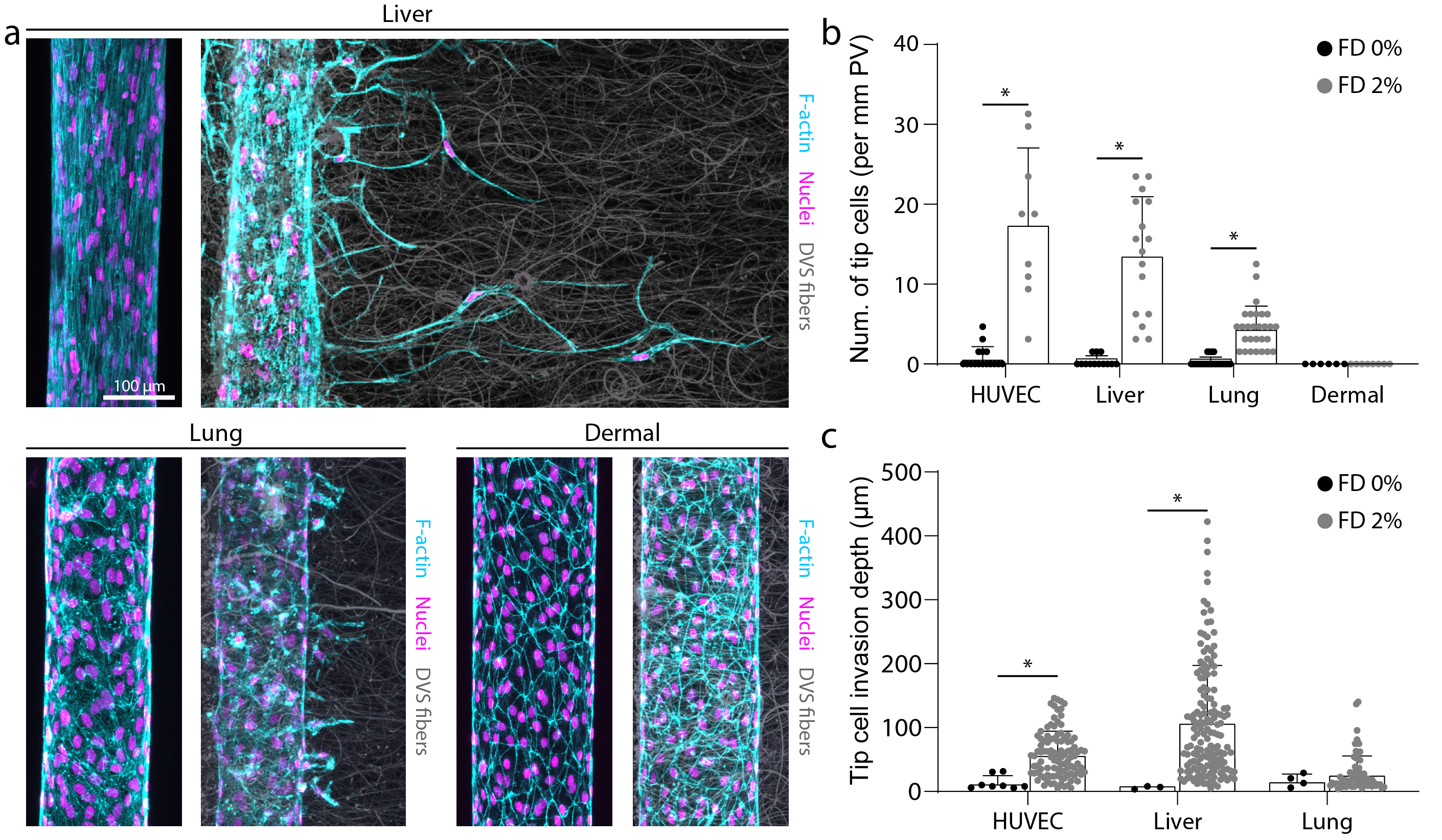


**Supplementary Figure 2: Heightened fiber density promotes ATEC formation from liver and lung but not dermal endothelial cell-lined microvessels. a)** Representative images (max intensity projections) of microvessels formed from liver, lung, or dermal endothelial cells cultured over 4-days in FD 0% or FD 2% hydrogels. Nuclei (magenta), F-actin (cyan), DVS fibers (grayscale). **b-c)** Corresponding quantification of number of tip cells and tip cell invasion depth. All data presented as mean ± std.; * indicates a statistically significant comparison with p < 0.05 (two-sided student’s t-test). Experiment was conducted with HUVECs.


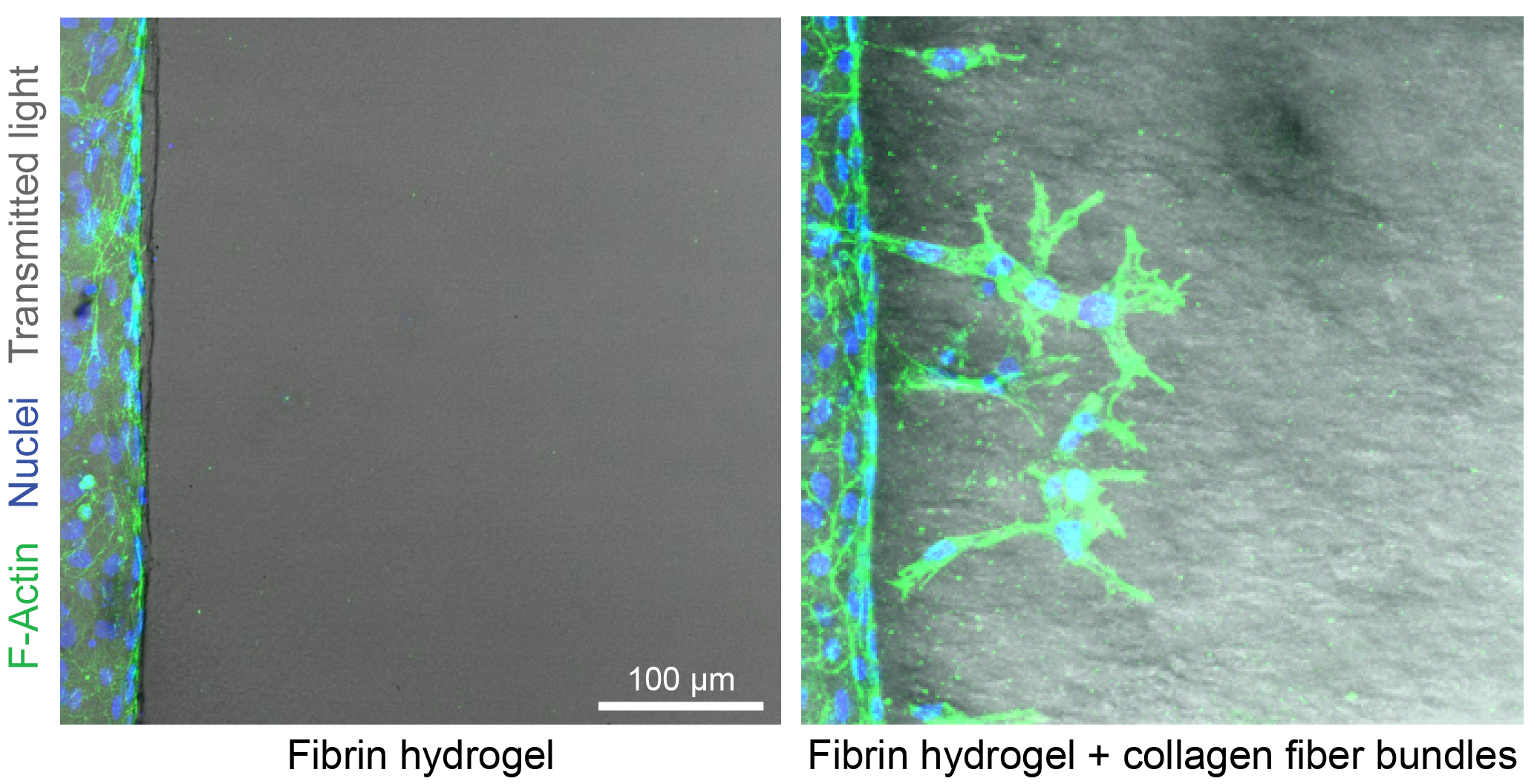


**Supplementary Figure 3: Type I collagen microfibers promote ATEC formation.** HUVEC microvessels within fibrin (10 mg/ml) hydrogel controls (left) or fibrin (10 mg/ml) supplemented with micrometer-scale collagen microfibers after 6 days of culture. Experiment was conducted with HUVECs.


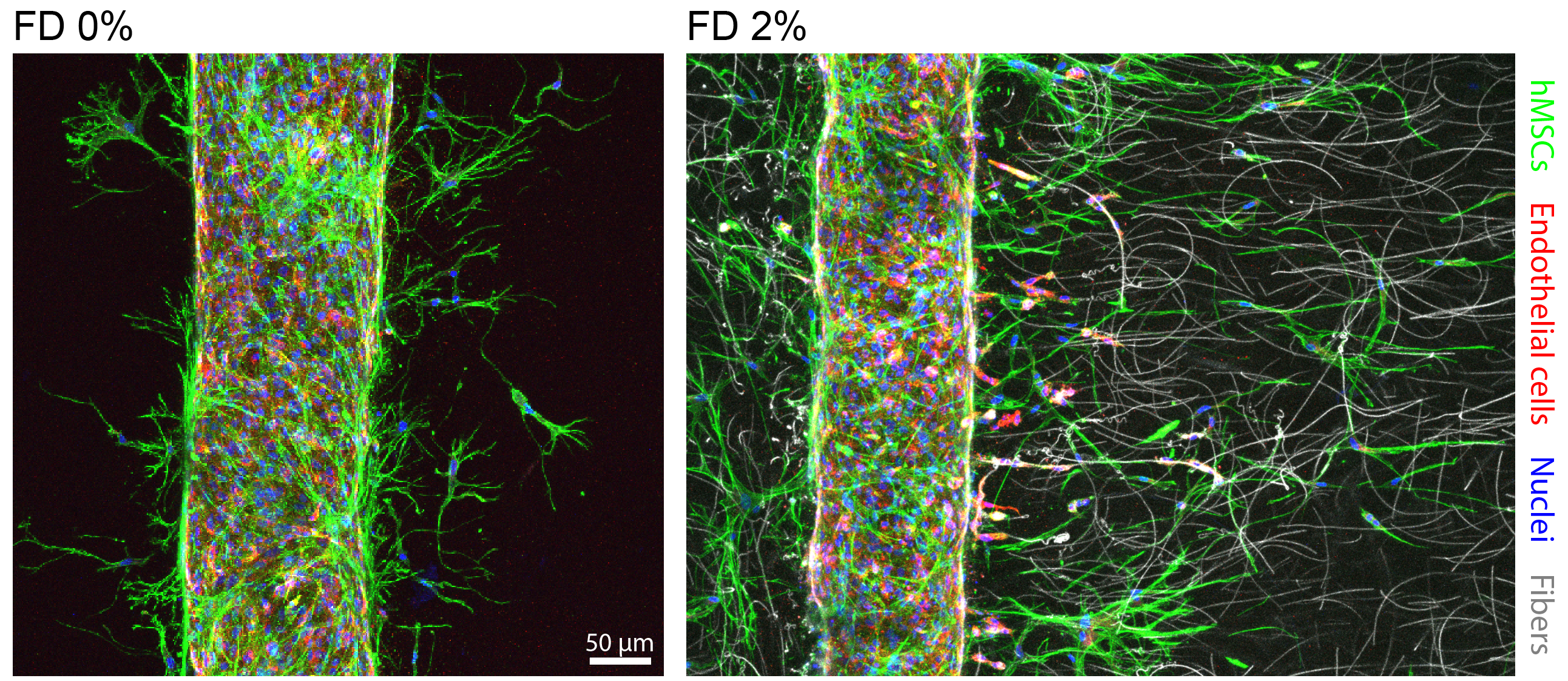


**Supplementary Figure 4: Heightened fiber density promotes ATEC formation despite the inclusion of pericyte-like hMSCs.** Representative images (max intensity projections) of hMSCs and endothelial cells cultured over 4-days in FD 0% control or FD 2% fibrin hydrogels. Experiment was conducted with HUVECs and hMSCs.


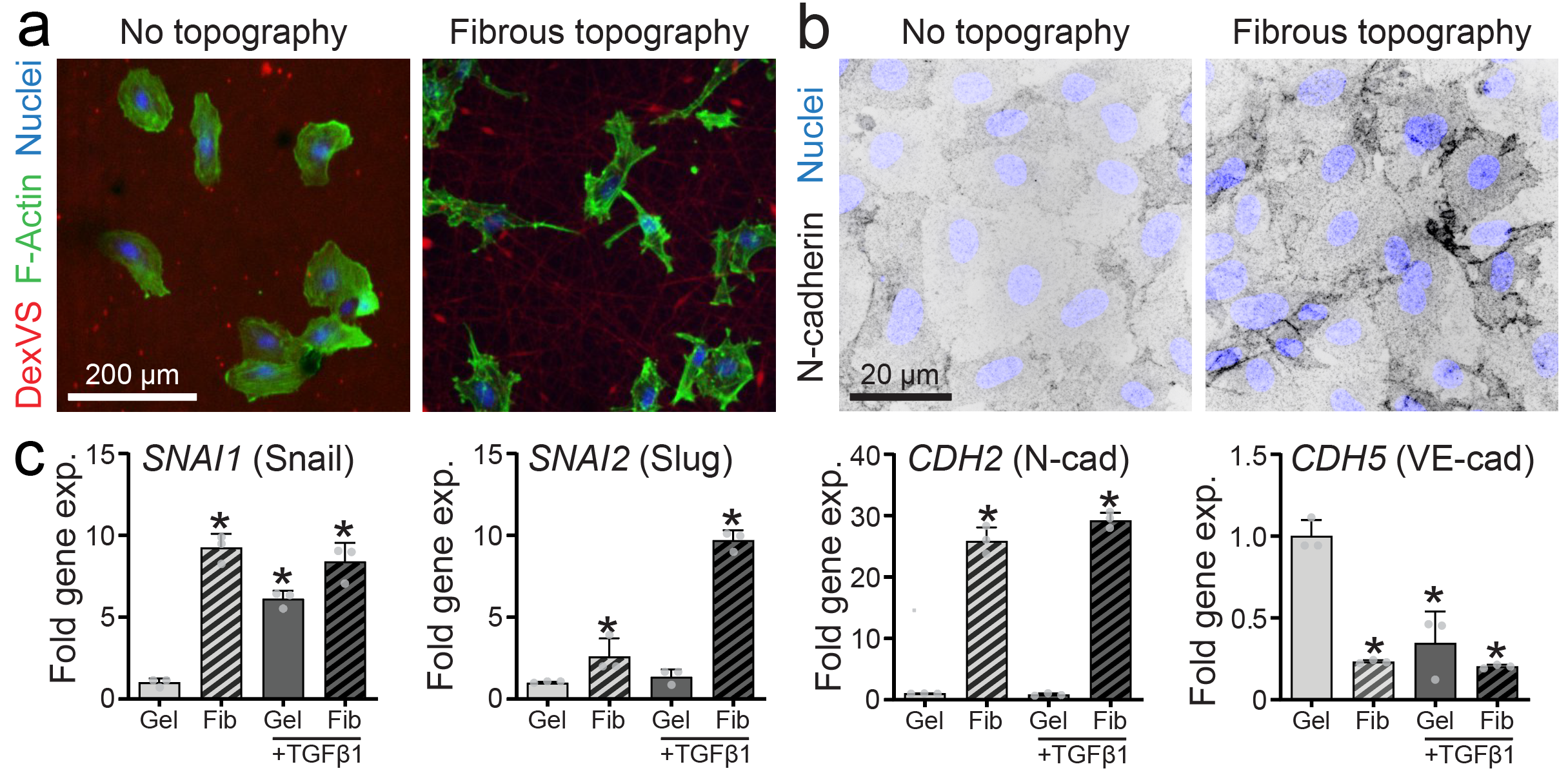


**Supplementary Figure 5: Fibrous topography and TGF-β drive EndMT.** **a)** Representative images (max intensity projections) of ECs seeded sparsely on flat DexVS hydrogels lacking topography and electrospun DexVS fibers, both functionalized with RGD. **b)** Immunostaining of N-cadherin of densely plated ECs. Note: intensity scale for N-cadherin in grayscale is inverted. **c)** Relative gene expression of sparsely plated ECs with or without exogenous TGF-β1 (5 ng/ml). Gene expression values normalized to flat DexVS gel substrates without TGF-β1 treatment. All data presented as mean ± std.; * indicates a statistically significant comparison with P<0.05 (one-way ANOVA with Tukey’s post hoc test). Representative data from N=3 replicate experiments. All experiments were conducted with HUVECs.


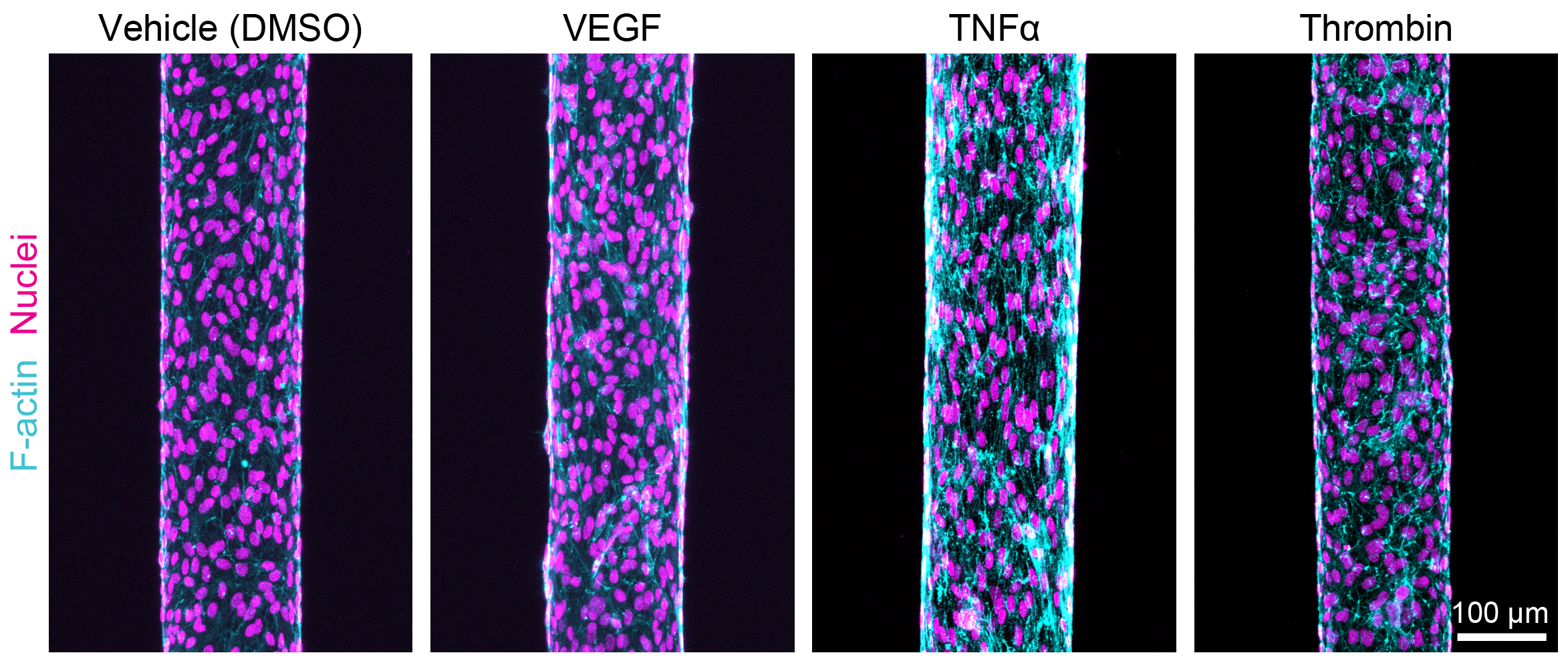


**Supplementary Figure 6: Permeability agonists do not promote ATEC formation in the absence of matrix fibers.** Representative maximum intensity projection images of microvessels within pure fibrin hydrogels (FD 0%) treated with vehicle (1:1000 DMSO), VEGF (50 ng/ml), TNFα (50 ng/ml), or thrombin (2 U/ml) after 4 days of culture along. Representative images from N=3 replicate experiments. All experiments were conducted with HUVECs.


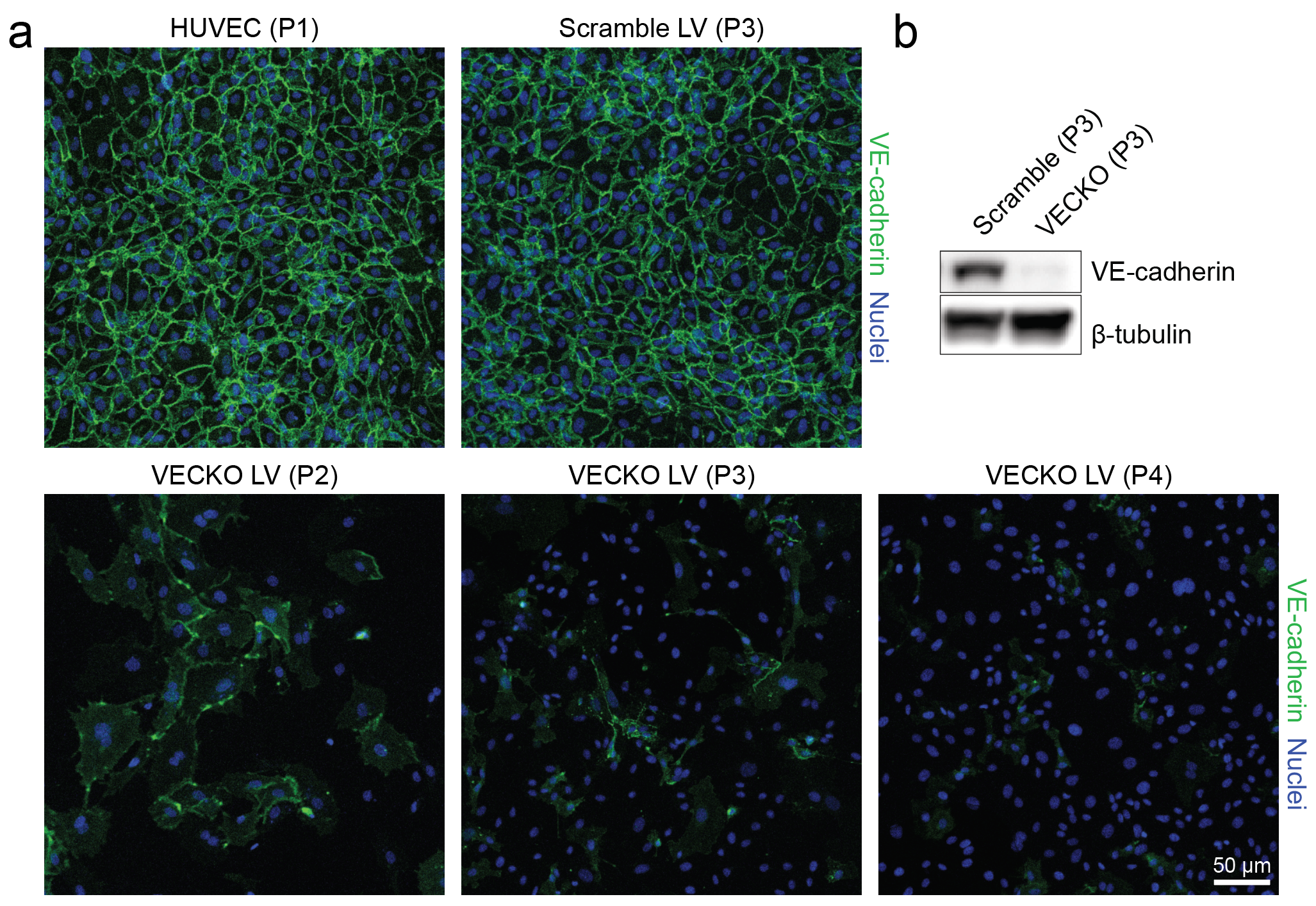


**Supplementary Figure 7: Confirmation of CRISPR-mediated VE-cadherin KO by immunofluorescence and Western blot. a)** Confocal images of HUVECs plated on tissue culture plastic immunostained for VE-cadherin including initial HUVECs (passage 1) prior to lentiviral transduction (top left), CRISPR scramble lentiviral control at passage 3 (top middle), and after CRISPR VE-cadherin KO (VECKO) lentiviral transduction across passages 2-4 (bottom row). **b)** Western blot of CRISPR scramble control and VECKO HUVECs at P3. Experiment was conducted with HUVECs.

**
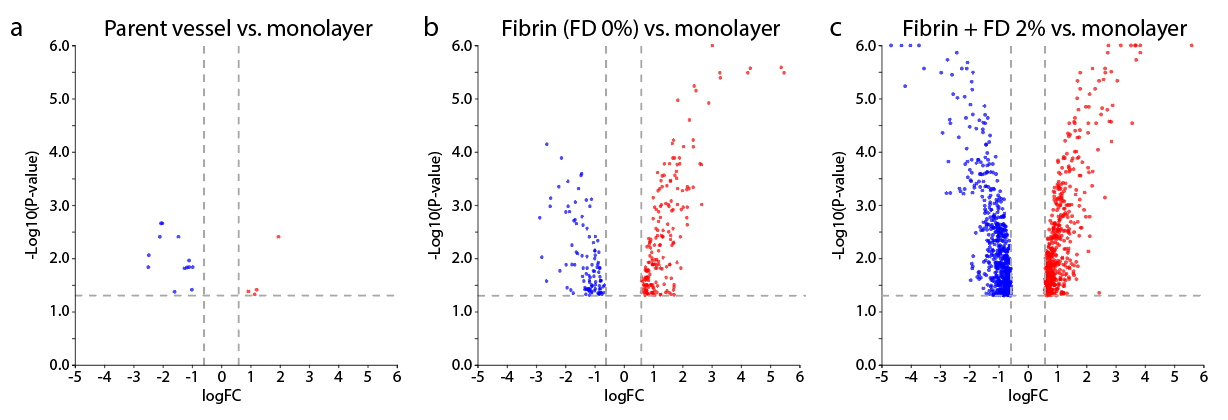
**

**Supplementary Figure 8: Volcano plots showing differentially expressed genes upon comparing HUVECs in parent vessels, control fibrin (FD 0%), or fibrin + FD 2% to monolayers cultured in 2D on fibrin.** Experiments was conducted with HUVECs.


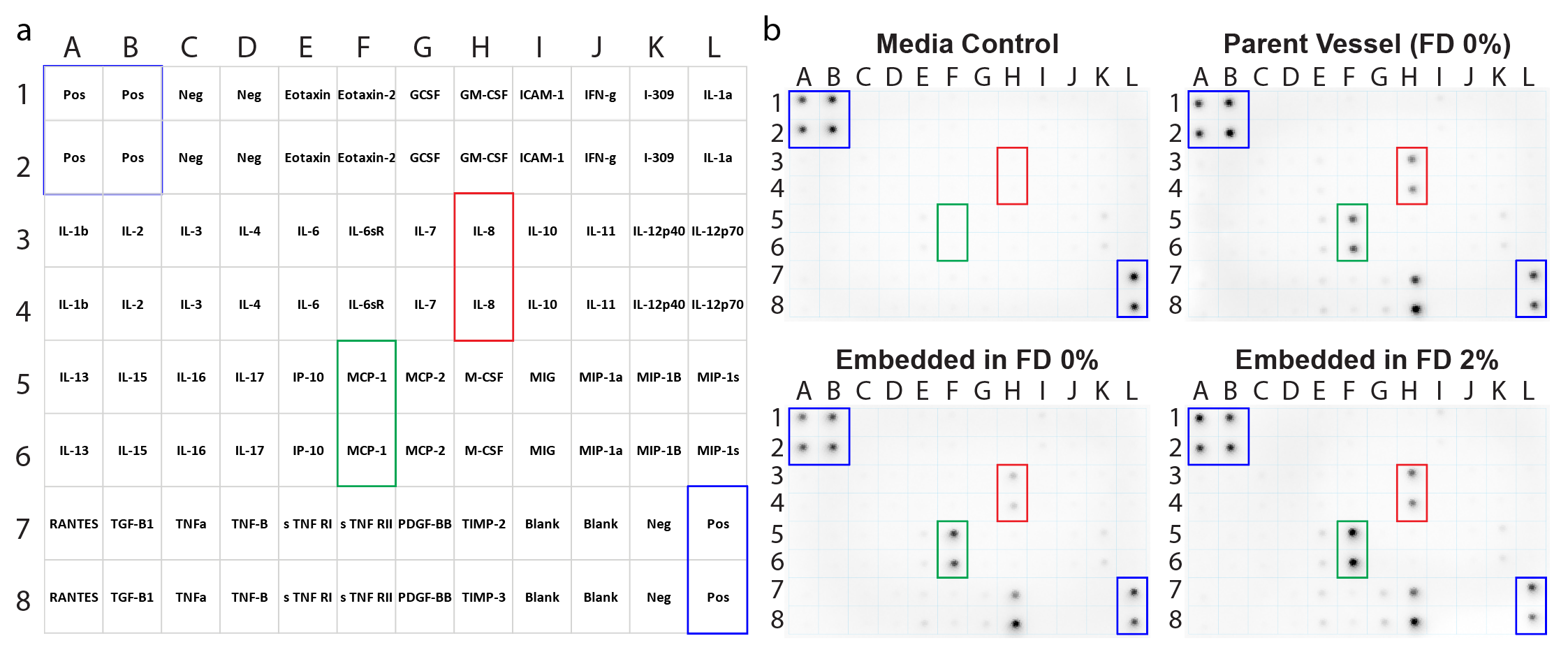


**Supplementary Figure 9: Increased secretion of IL-8 and MCP-1 from dispersed ECs engaging matrix fibers. a)** Layout of R&D Systems Proteome Profiler cytokine immunoblot array. **b)** Intensity-based response of captured proteins from media control, or conditioned media collected from ECs cultured as a parent vessel or embedded in fibrin hydrogel controls (FD 0%) or with FD 2%. Experiments was conducted with HUVECs.

**
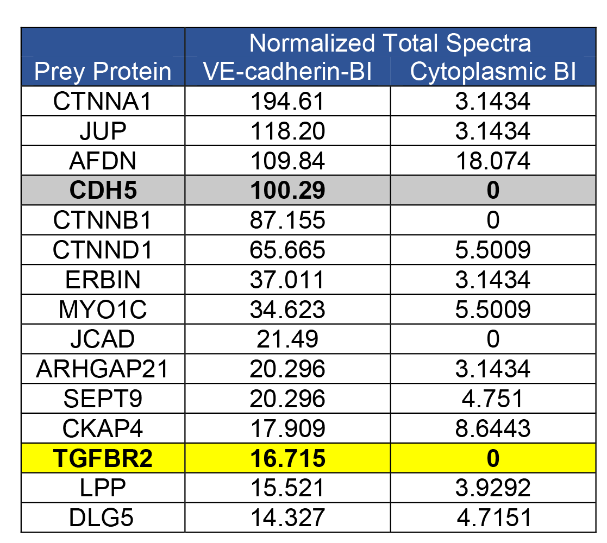
**

**Supplementary Figure 10: Top 15 VE-cadherin (CDH5) interactors identified via BioID and mass spectrometry analysis.** “VE-cadherin-BI” denotes VE-cadherin with fusion-tagged Bio ID construct (plasmid design shown in Figure 6A). The **“**Cytoplasmic BI” background control denotes the same construct expressing biotin ligase but without VE-cadherin inserted. Experiment was performed with hMVECs.


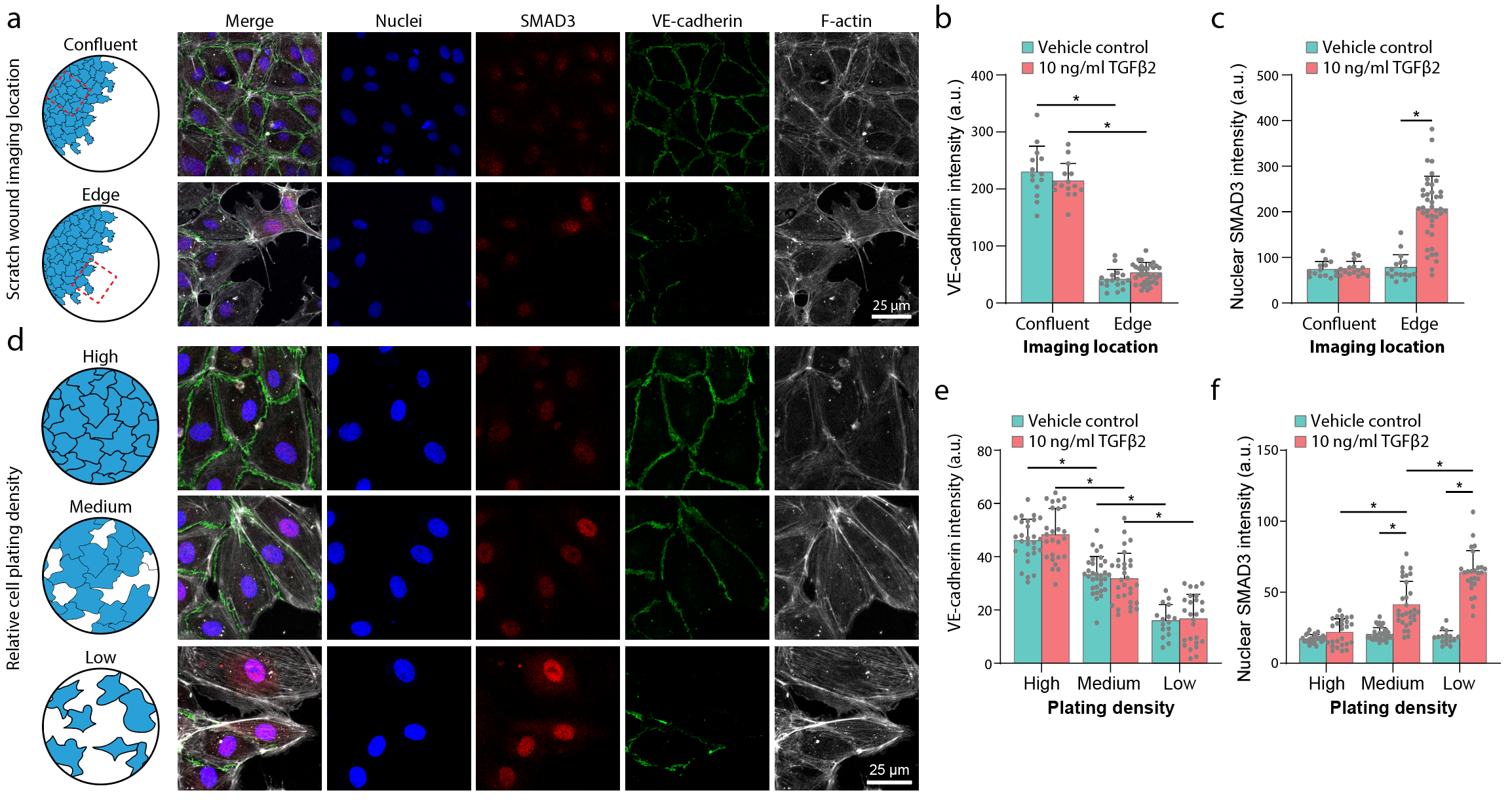


**Supplementary Figure 11: Local cell density and resulting VE-cadherin localization to AJs correlates with TGFβ2-induced nuclear localization of SMAD3.** **a)** Images from confluent vs. scratch wound edge regions within the same sample treated with TGFβ2 (10 ng/ml) for 1 day. **b-c)** Corresponding quantification of VE-cadherin intensity and nuclear SMAD3 localization in confluent vs. scratch regions treated with or without 10 ng/ml TGFβ2. **d)** HUVEC monolayers on fibronectin-coated glass seeded at relatively low, medium, or high densities to modulate local cell density, treated with 10 ng/ml TGFβ2 for 1 day. **e-f)** Corresponding quantification of VE-cadherin intensity and nuclear SMAD3 localization in as a function of plating density and treatment with or without 10 ng/ml TGFβ2. All data presented as mean ± std.; * indicates a statistically significant comparison with P<0.05 (one-way ANOVA). All images: nuclei (blue), SMAD3 (red), VE-cadherin (green), F-actin (white). Note: Images for vehicle controls are not shown due to observing only non-specific background levels of SMAD3 immunostaining. All experiments were conducted with HUVECs.


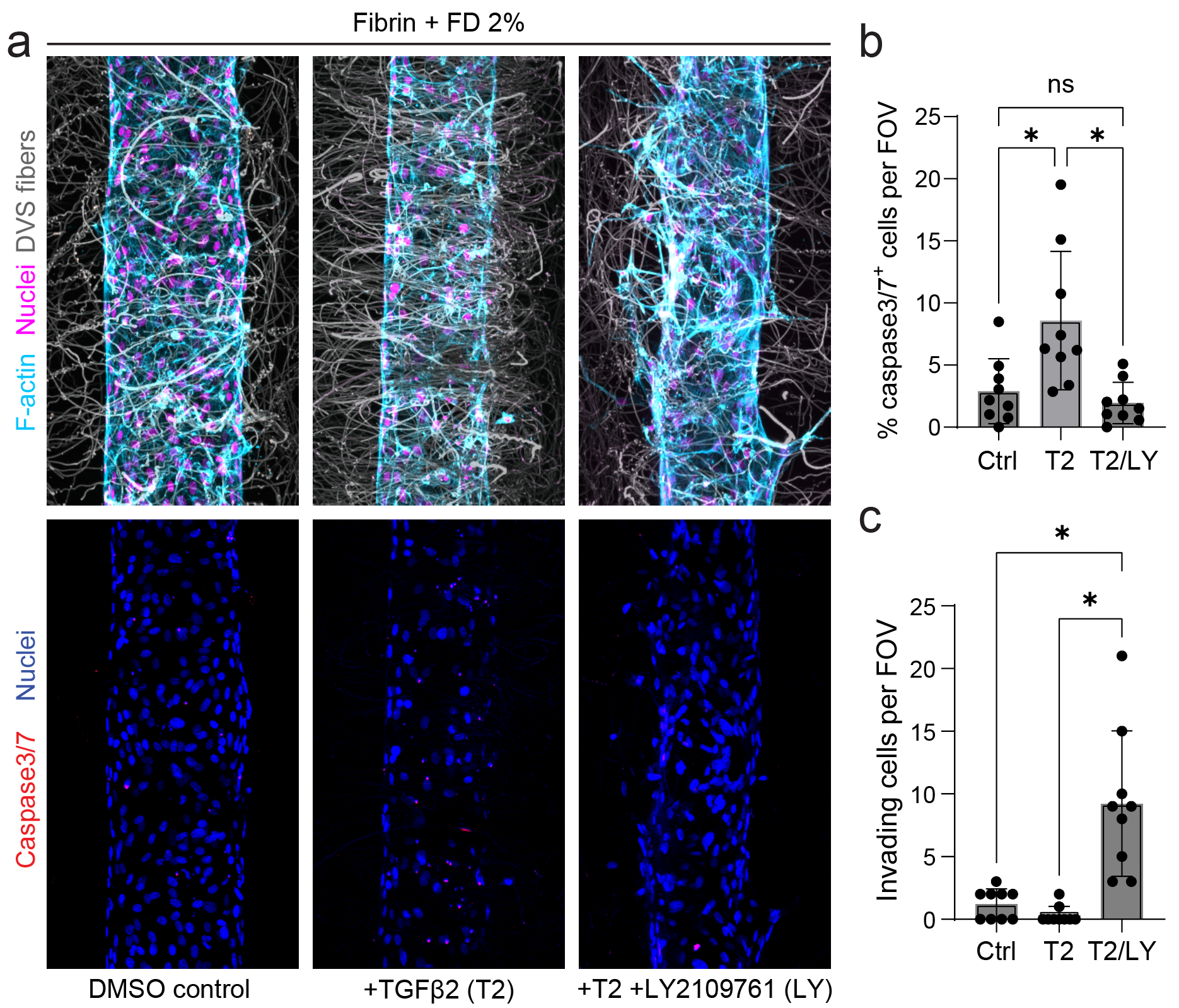


**Supplementary Figure 12: TGF-β inhibition via blockade of ALK5 (TGF-β type I receptor) abrogates EC apoptosis and leads to an increase in invasion. a)** Representative images (maximum intensity projections) of microvessels in FD2% hydrogels after 4 days of culture in the presence of TGF-β2 (10 ng/ml). LY2109761 (10 µM) or DMSO (1:1000 dilution) was added to culture media beginning on day 1. Top row: F-actin (cyan), nuclei (magenta); bottom row: nuclei (blue), activated caspase3/7 (red). Quantification of caspase3/7^+^ cells **(b)** and the number of cells invading from the parent vessel into the surrounding matrix **(c)** per FOV. N=3 devices each with 3 FOV per device over a single experiment. All data presented as mean ± std.; * indicates a statistically significant comparison with P<0.05 (one-way ANOVA with Tukey’s post hoc test). Experiment was conducted with HUVECs.
